# New insights into viral threats in soybean (*Glycine max*) crops from Bangladesh, including a novel crinivirus

**DOI:** 10.3389/fmicb.2025.1523767

**Published:** 2025-02-18

**Authors:** Mst. Fatema Khatun, Myeonghwan Kwak, Minhyeok Kwon, Md. Motaher Hossain, Eui-Joon Kil

**Affiliations:** ^1^Department of Plant Medicals, Andong National University, Andong, Republic of Korea; ^2^Agricultural Science and Technology Research Institute, Andong National University, Andong, Republic of Korea; ^3^Department of Entomology, Bangabandhu Sheikh Mujibur Rahman Agricultural University, Gazipur, Bangladesh; ^4^Department of Plant Pathology, Bangabandhu Sheikh Mujibur Rahman Agricultural University, Gazipur, Bangladesh

**Keywords:** mixed infections, high-throughput sequencing (HTS), crinivirus, novel virus, phylogenetic analysis

## Abstract

Soybean (*Glycine max*) crops in Bangladesh face significant challenges due to viral diseases, exacerbated by the hot and humid conditions that favor virus and vector proliferation. The emergence of novel or resurgent viruses can jeopardize soybean production because of the difficulties in identifying and characterizing these pathogens. In addition, detecting and characterizing new plant viruses demands considerable resources and commitment. This study examines soybean plants, collected from research fields in Gazipur, Bangladesh, between February 2022 and January 2023, that exhibited green or yellow mosaic, leaf wrinkling, and mild yellowing symptoms. Utilizing high-throughput sequencing, we examined the virome in soybean leaves. Our findings revealed the presence of three RNA viruses—*Potyvirus phaseovulgaris* (bean common mosaic virus, BCMV), *P. phaseoli* (bean common mosaic necrosis virus, BCMNV), a novel crinivirus—and one DNA virus, *Begomovirus vignaradiataindiaense* (mungbean yellow mosaic India virus, MYMIV). This is the first identification of the novel crinivirus, soybean mild yellows Bangladesh virus (SMYBV), in soybean. We assembled four viral genome sequences for phylogenetic analysis. The novel crinivirus is closely related to the criniviruses CCYV, LCV, and PloACV. BCMV and BCMNV exhibited high sequence similarity with a Bangladeshi isolate in the common bean, which indicated the continued spread of BCMV and BCMNV present in Bangladesh. Mixed infections with MYMIV-SMYBV, MYMIV-BCMV-BCMNV, and MYMIV-BCMNV were detected in soybean samples through RT-PCR and PCR.

## 1 Introduction

Soybean (*Glycine max* (L.) Merrill.) is one of the most important crops worldwide. It is used extensively to produce oil, food, animal feed (containing approximately 40% protein), and biofuels (Pagano and Miransari, 2016; Campos et al., 2014; Masuda and Goldsmith, 2009). In Bangladesh, soybeans have gradually gained popularity as a cash crop, particularly around the southern belt of the country (Noakhali, Lakshmipur, Chandpur, Barisal, and Bhola districts). However, in recent years, viral infections have begun to limit soybean yield (Miah and Mondal, 2017).

Over 100 viruses that infect soybeans have been identified, of which at least 46 have been detected in naturally occurring field infections (Hill and Whitham, 2014; Anderson et al., 2017). Among these reported viruses, the soybean mosaic virus (SMV) has spread globally, threatening soybean production worldwide (Hajimorad et al., 2018). Soybean production is threatened by novel and emerging viruses, in addition to well-known and established viruses (Campos et al., 2014; Hill and Whitham, 2014; Stuckey et al., 1982; Akanda et al., 1991; Giesler and Ziems, 2006; Harrison et al., 2005; Lim et al., 2014; Zhou et al., 2014; Elmore et al., 2022). High-throughput sequencing (HTS) enables the identification of both known and novel plant viruses within a short period. It can be applied for the identification and characterization of viruses, subsequently helping with their management (Cao et al., 2021; Liao et al., 2021; Zhang et al., 2021).

The genus *Crinivirus*, part of the family *Closteroviridae* and the order *Martellivirales*, comprises viruses primarily transmitted by whiteflies in a semi-persistent manner (ICTV, 2023; Tzanetakis et al., 2013). The global spread of whitefly vectors into temperate regions has amplified the agricultural significance of criniviruses as major plant pathogens [Fuchs et al., 2020; EFSA Panel on Plant Health (PLH), 2013]. Criniviruses have genomes comprising two linear, positive-sense, single-stranded RNAs, totaling 15.6–17.9 kb. Although genome organization is similar among the species of this genus, there are also apparent differences. In the majority of criniviruses, the genome is bipartite; however, the potato yellow vein virus has a tripartite genome (ICTV, 2023; Fuchs et al., 2020). All these molecules are required to cause infection and are individually encapsulated. RNA1 of criniviruses encodes three ORFs, with the 5′ region containing conserved motifs for papain-like proteinases, methyltransferases, and helicases. A (+1) ribosomal frameshift generates ORF1b, encoding an RNA-dependent RNA polymerase (RdRp) motif. Like other members of the *Closteroviridae* family, the ORF1a and ORF1b products form viral polymerases. The function of the 3’ ORF in RNA1, resembling the citrus tristeza virus ORF2 in size and location, remains unclear (Hull, 2002; Livieratos et al., 2004). RNA2 contains seven ORFs, organized similarly to the 3′ half of the closterovirus genome, with exceptions such as the absence of an additional 9-kDa ORF (ORF 2/4) upstream of the coat protein gene and rearrangements in the major and minor coat protein ORFs (ORFs 2/5 and 2/6) (ICTV, 2023). Downstream of the coat protein module, variations in the number and organization of ORFs are also observed among crinivirus species. As of 2023, the genus *Crinivirus* encompasses 14 recognized species, most of which are transmitted by whiteflies of the genera *Bemisia* and *Trialeurodes* in a semi-persistent manner, highlighting their role in the epidemiology of crinivirus-associated diseases (Liang et al., 2022; ICTV, 2023).

According to the International Committee on Taxonomy of Viruses (ICTV, 2023), *Potyvirus* is the largest among the 12 recognized genera within the family *Potyviridae.* The genome of potyviruses consists of a single-stranded, linear, positive-sense RNA of approximately 10 kb, encapsidated within flexuous, rod-shaped filaments primarily composed of a helically arranged coat protein (CP). Currently, the genus *Potyvirus* includes over 150 species, with several newly classified species added in recent years (ICTV, 2023; Inoue-Nagata et al., 2022). The viral genome encodes a single open reading frame (ORF) that is translated into a large polyprotein precursor of ~350 kDa, which is processed by virus-encoded proteases into 10 functional proteins (Ivanov et al., 2014; Chung et al., 2008). Among the notable plant RNA viruses, *Potyvirus phaseovulgaris* (bean common mosaic virus, BCMV) and *P. phaseoli* (bean common mosaic necrosis virus, BCMNV) belong to the genus *Potyvirus* within the family *Potyviridae*. These viruses are aphid-transmitted in a non-persistent manner and can also be seed-transmitted in common beans, with transmission efficiency reaching up to 80%, depending on the cultivar and strain (Drijfhout and Morales, 2005; Shankar et al., 2010). BCMV and BCMNV are among the most destructive pathogens affecting common bean production, causing common bean mosaic disease and associated yield losses. They have been reported in over 57 countries and regions, emphasizing their global importance (Chiquito-Almanza et al., 2017; Mwaipopo et al., 2018; Arli-Sokmen et al., 2016; Han et al., 2018).

The genus *Begomovirus*, the largest within the family *Geminiviridae*, comprises circular, single-stranded DNA viruses encapsulated in geminate particles. According to ICTV (2023), the genus contains over 445 species, reflecting its expansive diversity and ecological significance. Begomoviruses exhibit either monopartite or bipartite genomes. The bipartite genome structure consists of two DNA components, DNA-A and DNA-B, while monopartite genomes consist solely of DNA-A. DNA-A encodes essential proteins for replication (Rep), including replication initiator protein, encapsidation (coat protein), and regulation of gene expression, while also facilitating insect transmission. The DNA-B segment is critical for movement, encoding the movement protein (MP) and nuclear shuttle protein (NSP), which enable inter- and intracellular transport of the virus. Begomoviruses also express auxiliary proteins such as AV2 and AC4, which are involved in viral movement and modulation of the host cell cycle and defense mechanisms. These features distinguish begomoviruses from other geminiviruses. The monopartite begomoviruses often associate with betasatellites, which play roles in symptom expression and virulence (ICTV, 2023; Brown et al., 2015; Fiallo-Olivé et al., 2021; Briddon and Stanley, 2006).

Viruses have been detected in both symptomatic and asymptomatic plants. Many criniviruses have been shown to modify their symptoms by interfering with other viruses. Several studies have demonstrated that host-specific competition among crinivirus species affects the accumulation of other viruses within plants, thereby influencing symptom severity (Karyeija et al., 2000; Susaimuthu et al., 2008; Wintermantel et al., 2008). Such interactions between distantly related or unrelated viruses can lead to more severe disease outcomes, whereas single crinivirus infections often remain asymptomatic (Karyeija et al., 2000; Tzanetakis et al., 2004; Tzanetakis et al., 2006). Occasionally, two or more viruses may be simultaneously present in a single plant. Mixed viral infections in soybeans can alter virulence, symptoms, and disease transmission (Wintermantel et al., 2008; Wang et al., 2004; García-Cano et al., 2006). Mixed infections influence symptom emergence in two ways, resulting in either positive or negative interactions (Syller, 2012). Multiviral infections play a crucial role in viral evolution as they enable recombination, which can lead to the emergence of more severe viral strains or entirely new begomovirus species (Padidam et al., 1999; Sanz et al., 2000; Ribeiro et al., 2003). Significant progress is currently being made in understanding the interactions between criniviruses and their whitefly vectors (Chen et al., 2011; Stewart et al., 2010). This research direction is crucial as it may provide fundamental insights into crinivirus biology and pave the way for developing novel strategies to prevent diseases caused by various criniviruses (Kiss et al., 2013). However, effective methods for controlling criniviruses that cause severe crops infections remain limited. Therefore, studies focusing on virus control, particularly strategies to reduce virus transmission and identify resistance sources in both vectors and host plants, are urgently needed (Tzanetakis et al., 2013).

An alarming 43% of farmers in Bangladesh have faced challenges related to viral diseases affecting soybean cultivation, indicating significant economic impacts of virus diseases in the country (Islam and Khatun, 2022). The agricultural environment in Bangladesh confronts substantial obstacles due to its tropical climate, which promotes the spread of viruses and their carriers. Moreover, extensive pesticide use has led to the emergence of resistance among insect vectors, complicating control efforts and reducing crop yields. Consequently, an initiative was launched to identify and characterize soybean virus diseases. During the 2021–2022 growing season, soybean fields were scouted for virus-like disease symptoms at the BSMRAU research field in Gazipur, Bangladesh. Unusual symptoms, including green or yellow mosaic, leaf wrinkling, and mild yellowing in infected plants, were observed. More than a quarter of the plants displayed these mild yellowing symptoms (Authors’ observations). Similar symptoms were also noted in neighboring fields around BSMRAU in Gazipur. Given the distinct nature of the observed symptoms compared to common soybean virus diseases, symptomatic soybean leaves were collected for detection and characterization of the suspected virus(s). Total RNA was extracted from symptomatic soybean parts, cDNA libraries were prepared, and RNA sequencing was performed using HTS. A custom bioinformatic workflow was utilized to identify and assemble known and unknown virus genomes. Surprisingly, a novel crinivirus was identified and tentatively named “soybean mild yellows Bangladesh virus (SMYBV),” which was confirmed by RT-PCR and Sanger sequencing. Our research also unveiled the frequent occurrence of mixed viral infections within soybean fields.

## 2 Materials and methods

### 2.1 Sample collection, RNA extraction, and RNA-sequencing

**Figure 1:**
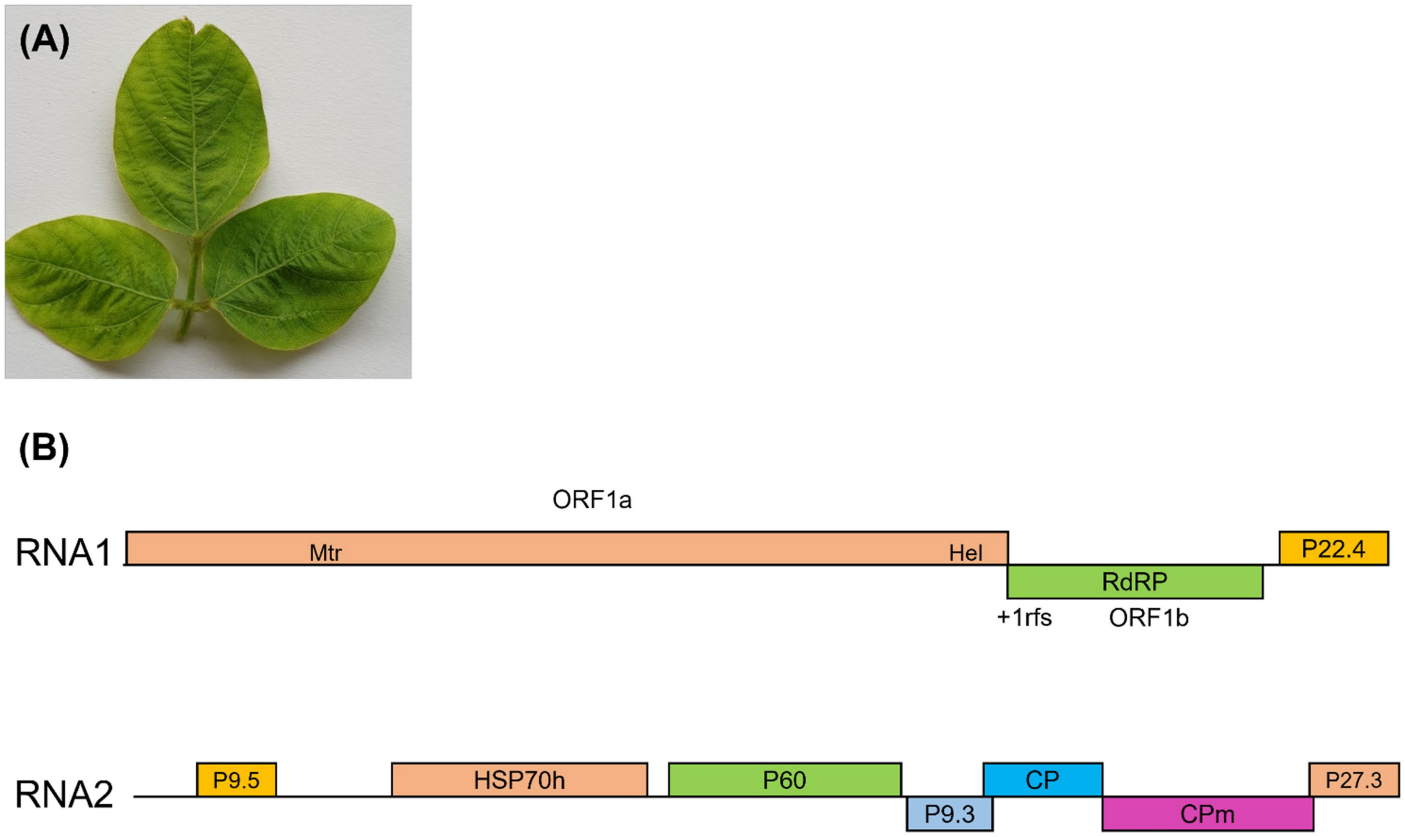
**(A)** Representative soybean leaves collected from research fields in Gazipur, Bangladesh, displaying virus-like symptoms, including green or yellow mosaic, leaf wrinkling, and mild yellowing. These samples were subjected to high-throughput sequencing (HTS) for virus detection. **(B)** A schematic of the soybean mild yellows Bangladesh virus (SMYBV) genome. The genomic RNA1 and RNA2 are presented by a line, with different ORFs. ORFs are shown above or below the line, indicating that they are found in different reading frames. On RNA1, the 1a/1b fusion polyprotein is produced through a +1 ribosomal frameshift site.

A total of 43 soybean leaf samples showing green or yellow mosaic, leaf wrinkling, and mild yellowing of viral infection were collected from the BSMRAU research field (collection coordinates, 23° 59′ 59.7876” N and 90° 25′ 12.9828″ E), Bangladesh, between February 2022 and January 2023 (Figure 1A; Supplementary Figure S1). To construct RNA-seq libraries, total RNA was extracted using the NucleoSpin RNA Plant kit (Macherey-Nagel, Düren, Germany) according to the manufacturer’s instructions. TruSeq Stranded Total RNA with Ribo-Zero Plant (Illumina, San Diego, CA, United States) was used for library construction. The quantity and quality of the total RNAs and libraries were estimated using a NanoPhotometer NP80 (Implen, München, Germany) and 4,200 TapeStation (Agilent Technologies, Santa Clara, CA, USA). Deep sequencing of libraries was performed using the Illumina NovaSeq 6,000 platform (Macrogen, Seoul, Korea).

### 2.2 Bioinformatic analysis

**Figure 2:**
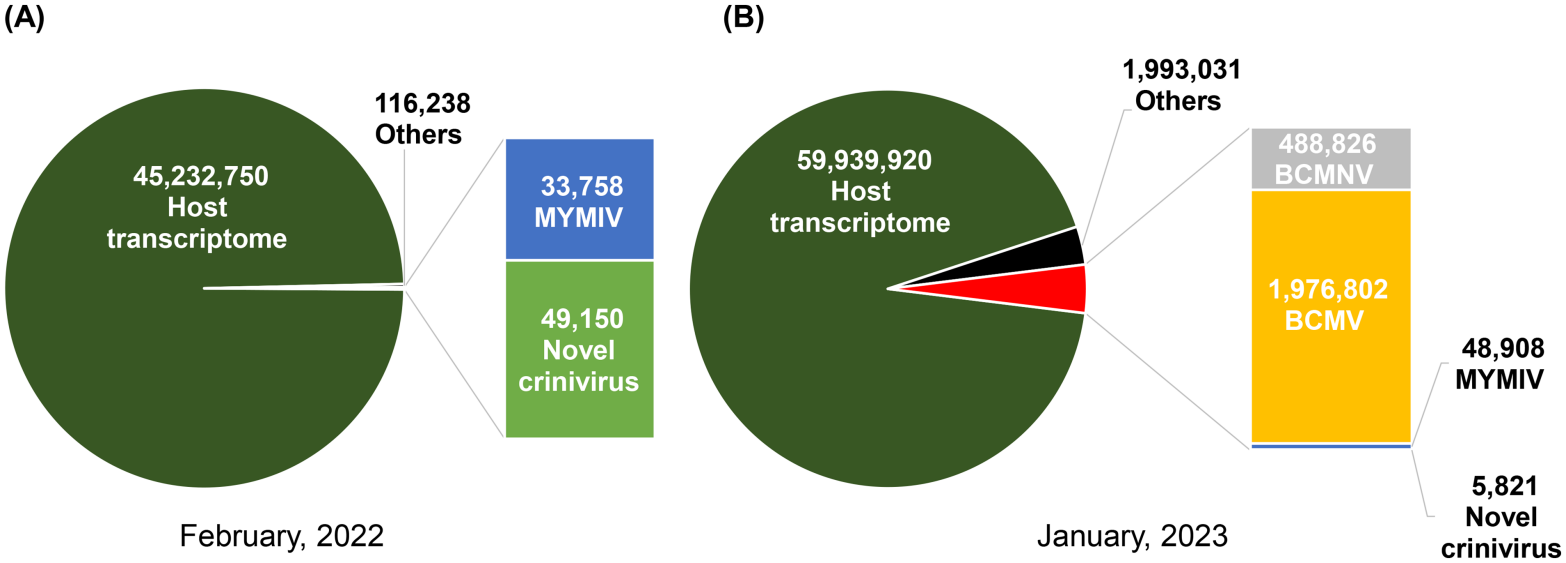
Pie charts illustrating the distribution of identified viruses, categorized by the number of viral reads in February 2022 **(A)** and January 2023 **(B)**.

The CLC Genomics Workbench software package (version 22.0.1, QIAGEN, Hilden, Germany) was used to analyze RNA-seq data from two different years, 2022 and 2023. The “Trim Reads” program within CLC software removed low-quality reads and adapters of output raw reads. The host genome of (*Glycine max*) was downloaded from the NCBI Genome database, the host genome was mapped to trimmed reads, and unmapped reads were collected (Figure 2). The CLC workbench was used to assemble the reads *de novo*. The assembled contigs were searched against the NCBI nucleotide and protein databases using default parameters for BLASTn and BLASTp/tBLASTx. We used NCBI BLASTn to explore the virus database, extracting contigs from the tBLASTx results with E-values falling within the range of 0 to xe-10. The novel virus was classified according to the species demarcation criteria of ICTV (2023).

### 2.3 Confirmation of viruses using RT-PCR and PCR

RT-PCR was performed to confirm the presence of a novel crinivirus (Supplementary Table S1). The NCBI Primer-BLAST tool was used to design a primer sequence based on the assembled contigs (https://criniviruses.ncbi.nlm.nih.gov/tools/primer-blast/; Table 1). Primer pairs for RNA1 and RNA2 segments were used in RT-PCR under the following conditions: 30 min at 50°C, 5 min at 95°C, 35 cycles of 30 s at 95°C, 30 s at 58°C and 60°C, respectively, and 1 min at 72°C, followed by 5 min at 72°C. DNA-A and DNA-B primer pairs were used in PCR under the following conditions: 5 min at 95°C, followed by 35 cycles of 30 s at 95°C, 30 s at 59°C, and 58°C, respectively, and 1 min at 72°C, with a final extension at 72°C for 5 min (Table 1). BCMNV and BCMV were used in RT-PCR under the following conditions: 30 min at 50°C, 5 min at 95°C, 35 cycles of 30 s at 95°C, 30 s at 59°C and 60°C, respectively, and 1 min at 72°C, followed by 5 min at 72°C (Table 1). Electrophoresis of the PCR products on a 1% agarose gel was carried out (Supplementary Figure S3). The gel was purified using the Expin SV gel and PCR clean-up system (GeneAll, Seoul, Korea). The PCR products were subcloned into the pGEM-T Easy vector (Promega, Madison, WI, United States) and sequenced via Sanger sequencing (Macrogen).

**Table 1:** Information on primers used for RT-PCR and PCR.

Primer name	Segments	Primer seq (5′ – 3′)	Product size (bp)/Tm (°C)
Crinivirus_RNA1F1	RNA1	AACGGTTTGCACTGATTGGC	979/58
Crinivirus_RNA1R1		ATCTGTTCGTGACGCAGTGT	
Crinivirus_RNA1f1		TTATCATCGTCCCGTGCTGG	808/60
Crinivirus_RNA1r1		GCACCAGGAGGTTGTGGTAA	
Crinivirus_RNA2F1	RNA2	CCTTCTGTGTCTGCCACTGT	532/60
Crinivirus_RNA2R1		CTGGTGATGTGGTTGTCGGA	
Crinivirus_RNA2f1		GGGATTCTTCACCCGTCGTC	324/61
Crinivirus_RNA2r1		ACGCCTTCCCCTCGAAATG	
MYMIV_DNA_AF1	DNA-A	TACAACCACCAAGAGGCAGC	431/59
MYMIV_DNA_AR1		CAAGCAGGGTCCAGAATGGT	
MYMIV_DNA_Af1		ACCATTCTGGACCCTGCTTG	355/61
MYMIV_DNA_Ar1		CCTGGTCCCAAGTCCTCCTA	
MYMIV_DNA_BF1	DNA-B	ACCGCAATTATCGCACTCCT	447/58
MYMIV_DNA_BR1		ATAACTCCGCAAAGCAGGGT	
MYMIV_DNA_Bf1		GGTCCCTCTATTACGTGGCG	640/60
MYMIV_DNA_Br1		ACGTCCATCCCCGTTGATTC	
BCMNV_F1 BCMNV_R1	-	AGGGGGCTCAACAGATTTGG CCAGTTCCTTGTGCTTGTGC	311/59
BCMV_F1 BCMV_R1	-	GCCACTGATTTGGATGGGGA TCGCTCACAGAGGAGACAGA	662/60

### 2.4 Sequence analysis and phylogenetic reconstruction

The ORF Finder tool^1^ was used to predict open reading frames (ORFs) by translating the full-length genomic nucleotide sequences of each viral genomic RNA component into amino acid sequences. To identify homologous viral protein sequences, each protein was BLASTed against the NCBI’s non-redundant protein database (nr). Multiple sequences were aligned using the CLC Genomics Workbench software. The ‘Create Pairwise Comparison’ function in the CLC Genomics Workbench calculated pairwise identities of nucleotide and amino acid sequences among the newly confirmed viruses and member species. The pairwise nucleotide identity color distance matrix was calculated by SDT v1.2 (Supplementary Figure S4). Sequences of the member species of *Crinivirus* (ORF1a, ORF1a/b containing RdRp, HSP70h, CP, and CPm) were obtained from the ICTV and NCBI databases. Nucleotide sequences were compared to the NCBI nucleotide database using BLASTn, while amino acid sequences were compared to the protein database using BLASTp/tBLASTx. Aligned nucleotide and amino acid sequences and phylogenetic tree construction using the maximum likelihood method (1,000 bootstrap replicates) were conducted using MEGA 11.0 (Tamura et al., 2021; Felsenstein, 1985).

### 2.5 Seed transmission tests

Seed transmission tests were performed to evaluate the transmissibility of SMYBV and *Begomovirus vignaradiataindiaense* (mungbean yellow mosaic India virus, MYMIV) using seeds collected from Bangladesh in 2023. After 4 weeks of growth, two young leaflets from each soybean seedling were freshly harvested, and total RNA and DNA were extracted. The RNA from each plant was tested for the presence of the crinivirus (SMYBV) using RT-PCR, while DNA was analyzed for the begomovirus (MYMIV) using PCR.

### 2.6 Mechanical inoculation

Soybean and tobacco plants were grown under greenhouse conditions. Leaf tissue samples (0.5 g) from infected soybeans were homogenized in 1.5 mL potassium phosphate buffer (PBS, pH 7.4) and mechanically inoculated into three groups of soybean plants. To achieve this, 150 μL sap was rubbed onto the first true leaf, which had previously been dusted with carborundum powder. The total RNA was extracted 14 days after inoculation, and RT-PCR was performed to identify viral infections in soybean plants.

## 3 Results

### 3.1 Virus identification

**Figure 3:**
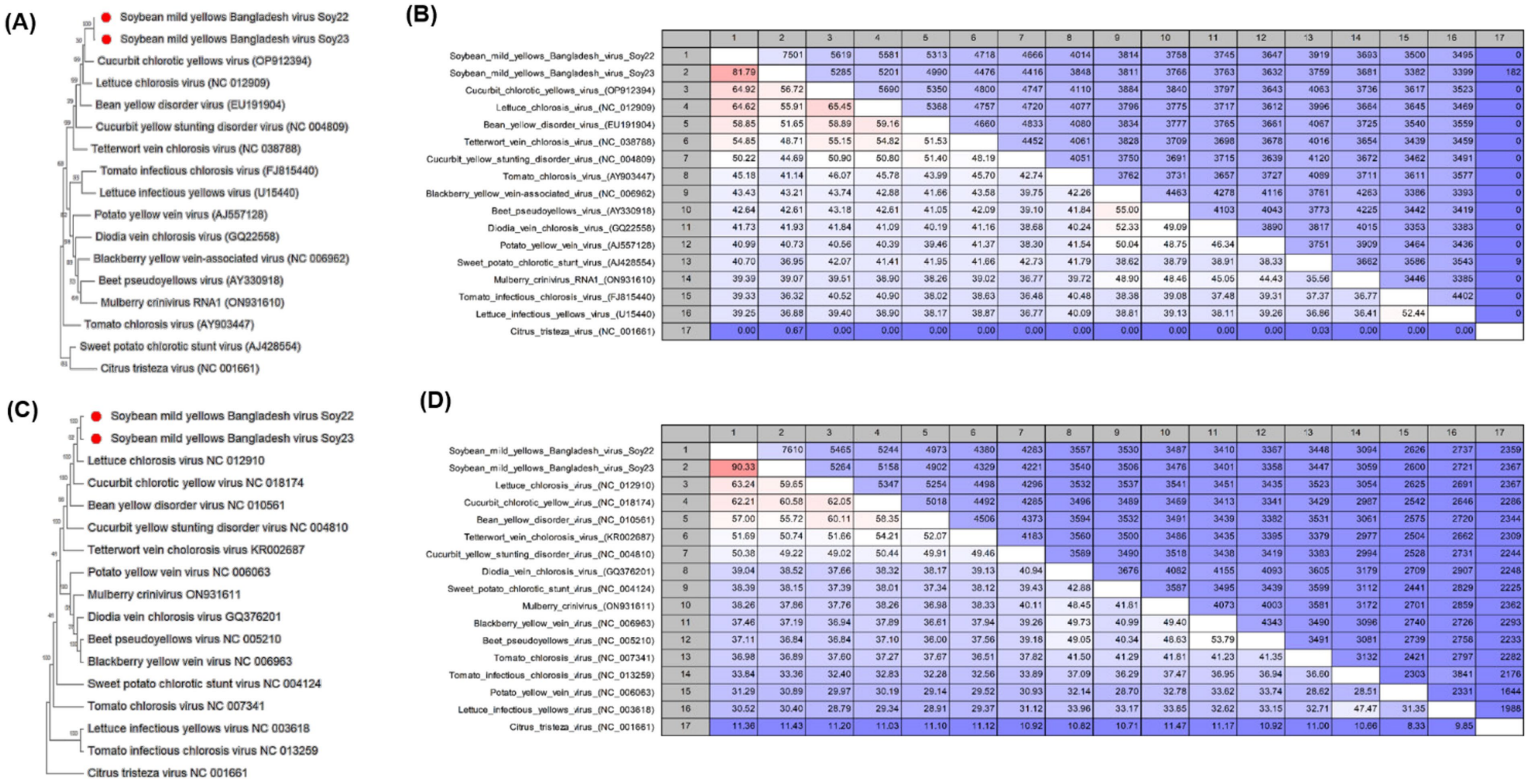
**(A,C)** Phylogenetic trees showing the relationship between soybean mild yellows Bangladesh virus (SMYBV, RNA1, and RNA2) and other criniviruses. The trees were constructed using the maximum likelihood method and the Jukes–Cantor model with 1,000 bootstrap replicates. Evolutionary analyses were conducted using Mega 11. GenBank accession numbers for each sequence are shown. **(B,D)** Pairwise comparisons between SMYBV and other complete crinivirus sequences were reported in the NCBI GenBank database using the CLC Genomics Workbench. In pairwise comparisons among the sequences, the upper level showed identity, whereas the lower level showed percentage identity.

We identified the viruses responsible for green or yellow mosaic, leaf wrinkling, and mild yellowing in soybean leaves (Figure 1A; Supplementary Figure S1) using RNA sequencing. The raw reads underwent analysis following the outlined workflow (Supplementary Figure S2). Following trimming, the number of reads obtained was 49,024,003 for the samples collected in 2022 and 66,170,397 for those collected in 2023. After filtering the BLAST results, it was observed that three distinct plant viral families are present: *Closteroviridae*, *Geminiviridae*, and *Potyviridae.* The sequences were subsequently deposited in the GenBank database with accession numbers (Table 2). In 2022, the analysis of HTS data using BLAST against the viral reference database revealed the presence of viruses. This analysis identified two contigs (8,465 nt and 8,168 nt) containing two distinct genomic RNA segments associated with members of the genus *Crinivirus*. In addition, two other contigs (2,746 and 2,634 nt) demonstrated similarities to DNA-A and DNA-B of MYMIV. Furthermore, the second set of HTS data in 2023 revealed the presence of two contigs associated with *Crinivirus* (8,459 nt and 8,176 nt) and two contigs linked to mungbean yellow mosaic India virus (MYMIV) (2,746 nt and 2,634 nt). In addition, a contig of BCMNV (9,619 nt) and a contig of BCMV (10,052 nt), both members of the *Potyvirus* genus, were identified (Table 2). Hence, the HTS analysis indicated that soybean plants in Bangladesh had been infected by four viruses, including a novel crinivirus (Figures 3A,C), along with variants of the known MYMIV, BCMNV, and BCMV. Based on these data, we hypothesized that the sequenced sample potentially harbored a novel crinivirus, which we have designated as the soybean mild yellows Bangladesh virus (SMYBV). The assembled complete genome sequences of each virus were subsequently deposited in the GenBank database, where they were assigned unique accession numbers (Table 2).

**Table 2:** Viruses were identified through HTS from *Glycine max* leaves.

	Family	Genus	Virus	Occurrence in Bangladesh	Consensus length (nt)	Accession number
2022	*Closteroviridae*	*Crinivirus*	Novel crinivirus segment RNA1	Novel	8,465 8,168	OR215422
			Novel crinivirus segment RNA2			OR215423
	*Geminiviridae*	*Begomovirus*	*Begomovirus vignaradiataindiaense* (mungbean yellow mosaic India virus, MYMIV) segment DNA-A	Reported	2,746 2,655	OR215424
			MYMIV segment DNA-B			OR215425
2023	*Potyviridae*	*Potyvirus*	*Potyvirus phaseoli* (bean common mosaic necrosis virus, BCMNV)	Reported	9,619	PQ811724
			*Potyvirus phaseovulgaris* (bean common mosaic virus, BCMV)	Reported	10,052	PQ811723
	*Geminiviridae*	*Begomovirus*	MYMIV segment DNA-A	Reported	2,746 2,634	PQ811721
			MYMIV segment DNA-B			PQ811722
	*Closteroviridae*	*Crinivirus*	Novel crinivirus segment RNA1	Novel	8,459 8,176	PQ811719
			Novel crinivirus segment RNA2			PQ811720

### 3.2 Virus genome confirmation and analyses

The two contigs corresponded to RNA1 and RNA2 of the soybean mild yellows Bangladesh virus (SMYBV), a novel crinivirus (Figure 1B). Subsequently, both genomic RNA segments were amplified with primers designed from the contig sequences (Table 1) and then sequenced. The resulting amplicons showed the expected target sizes (Supplementary Figure S3). Analysis of the complete genome sequence of SMYBV revealed that RNA1 exhibited the highest percentage identities at the nucleotide level with *Crinivirus lactucachlorosi* (lettuce chlorosis virus, LCV, NC_012909) and cucurbit chlorotic yellow virus (CCYV, OP912394), reaching 64.62 and 64.92%, respectively (Figure 3B). In addition, RNA2 showed similarities of 63.24 and 62.21% with LCV (NC_012910) and CCYV (NC_018174), respectively (Figure 3D). The nucleotide identities for RNA1 were 81.79% between SMYBV Soy22 and SMYBV Soy23, while for RNA2, the identity was 99.33% between SMYBV Soy22 and SMYBV Soy23. Significantly, both RNA1 and RNA2 segments were detected in a single sample from both the 2022 and 2023 collected samples.

RNA1 consisted of 8,465 nucleotides, featuring three open reading frames [ORF1a (60–6,023), 1b (6024–7,539), and P22.4 (7666–8,250)] that may encode proteins involved in virus replication and gene silencing suppression (Figure 1B). ORF1 was expressed through a + 1 ribosomal frameshift of ORF1a/1b, which contains the RNA-dependent RNA polymerase (RdRp), a pattern observed in other criniviruses. ORF1a encoded a protein consisting of 1,988 amino acids, demonstrating a 67.63% similarity to CCYV. Moreover, the ORF1a/b protein displayed a 71.12% similarity to the RdRp of CCYV (Table 3).

**Table 3:** Position and percentage identities of nucleotides and the amino acids of the *Crinivirus* from soybean.

RNA Segment	Gene	Nucleotide position	No. of amino acids	Percent identity with NCBI database	Nucleotide comparison (accession no.)	Amino acid comparison (accession no.)
RNA1	ORF1a	60–6,023	1988	71.33% nt and 67.63% aa	CCYV (MH477611)	CCYV (ATX62252)
	ORF1a/1b (RdRp)	60–7,539	2,493	72.26% nt and 71.12% aa	LCV (OQ377539)	CCYV (UXR26842)
	P22.4	7,666–8,250	195	26.74% aa	-	LCV (WIC44015)
RNA2	P9.5	262–519	86	84.16% nt and 61.36% aa	CCYV (MH477612)	LCV (QEM23779)
	HSP70h	1,536–3,206	557	75.85% nt and 87.79% aa	LCV (MN203150)	LCV (ASS35987)
	P60	3,365–4,918	518	72.71% nt and 75.58% aa	PloACV (ON181429)	PloACV (UQV97400)
	P9.3	4,900–5,139	80	71.37% nt and 68.35% aa	CCYV (OP912397)	CCYV (ATX62248)
	CP	5,235–5,987	251	78.65% nt and 75.29% aa	LCV (MK747245)	CCYV (AEN14595)
	CPm	5,987–7,411	475	67.64% nt and 64.93% aa	CCYV (MZ405664)	PloACV (UQV97403)
	p27.3	7,413–8,114	234	67.47% nt and 59.23% aa	PloACV (ON181429)	LCV (QHB15123)

* The meaning is as follows: “(nt)” nucleotide, “(aa)” amino acid. CCYV—cucurbit chlorotic yellow virus, LCV—crinivirus lactucachlorosi (lettuce chlorosis virus), PloACV—*Pueraria lobata*-associated crinivirus.

Closterovirus had two conserved domains associated with viral replication: the viral methyltransferase domain from the methyltransferase superfamily (Pfam01660), positioned between amino acid sites 532 and 849 (E-value = 0.0), and the RNA helicase domain from the viral helicase 1 superfamily (Pfam01443), situated between amino acid sites 1,690 and 1,953 (E-value = 7e-155) (Figure 1B). The putative RNA-dependent RNA polymerase (RdRp) associated with positive-sense single-stranded RNA viruses, cd23253, was coded by ORF1b, including 505 amino acids and positioned between amino acids 2,168 and 2,435 (E-value = 0.0). ORF3, 585 nucleotides in length, encoded a protein (P22.4) comprising 195 amino acids (E-value = 0.003). There was no substantial sequence similarity between ORF3 and equivalent proteins of other criniviruses. RNA2 comprised 8,176 nucleotides (NCBI accession number ON181429) and featured seven ORFs (Figure 1B). Small ORF1 may encode protein P9.5, which was located in the 5′-terminal region of RNA2. Within RNA2, several functional elements were encoded, including HSP70h (nt 1,536–3,206) housing a nucleotide-binding domain and viral heat shock protein Hsp90 homolog (pfam03225). In addition, a major coat protein (pfam01785 CP) was encoded between nucleotide positions 5,235 and 5,987, while a minor coat protein occupied positions 5,987 to 7,411. P60 (putative p60 protein) and P9.3 (hypothetical 9-kDa protein), alongside P27.3 (Table 3), were positioned between the HSP70h and CP ORFs.

Two contigs corresponding to the mungbean yellow mosaic India virus (MYMIV) were assembled from a total of 33,758 viral reads in 2022 and 48,908 reads in 2023 (Figure 2). The genome, amplified using the primers MYMIV_DNA_A2F/MYMIV_DNA_A2R and MYMIV_DNA_B2F/MYMIV_DNA_B2R (Table 1), was sequenced. The findings revealed a bipartite genome with circular DNA-A and DNA-B, measuring 2,746 nt and 2,655 nt, and 2,746 nt and 2,634 nt in length, respectively (Table 2). DNA-A exhibited a nucleotide sequence identity of 99.60%, while DNA-B showed a 98.57% identity with a known MYMIV. DNA-A consisted of six ORFs, including sense proteins AV1 (CP) and AV2, and antisense proteins AC1 (Rep), AC2 (TrAP), AC3 (REn), and AC4. DNA-B encoded two ORFs: BC1 (MP) in the virion sense and BV1 (NSP) in the complementary sense, as illustrated in Supplementary Figure S7. The intergenic region, also known as the common region (CR), contained a conserved stem-loop.

The genomes of BCMNV and BCMV were successfully assembled using HTS data. In 2023, BCMNV (488,826 viral reads) and BCMV (1,976,802 viral reads) were identified in soybean samples from Bangladesh; however, these viruses were not detected in the 2022 samples (Figure 2). These viruses are recognized as common bean pathogens and are known to infect common beans worldwide, not limited to Bangladesh. BLAST analysis revealed a complete sequence for both viruses and that sequence was 99% similar to Bangladesh isolates at both nucleotide and amino acid levels. The genomes of BCMV and BCMNV contain a large open reading frame (ORF) that is translated into a polypeptide. This polypeptide undergoes hydrolysis by three viral proteinases, resulting in the production of ten mature proteins: protein 1 (P1), a helper component-protease (HC-Pro), protein 3 (P3), a 6-kilodalton protein 1 (6 K1), a cylindrical inclusion (CI), a 6-kilodalton protein 2 (6 K2), a viral protein genome-linked (VPg), a nuclear inclusion a-protease (NIa-Pro), a nuclear inclusion b (NIb), and a coat proteins (Supplementary Figure S7).

### 3.3 Phylogenetic analysis and pairwise comparison

**Figure 4:**
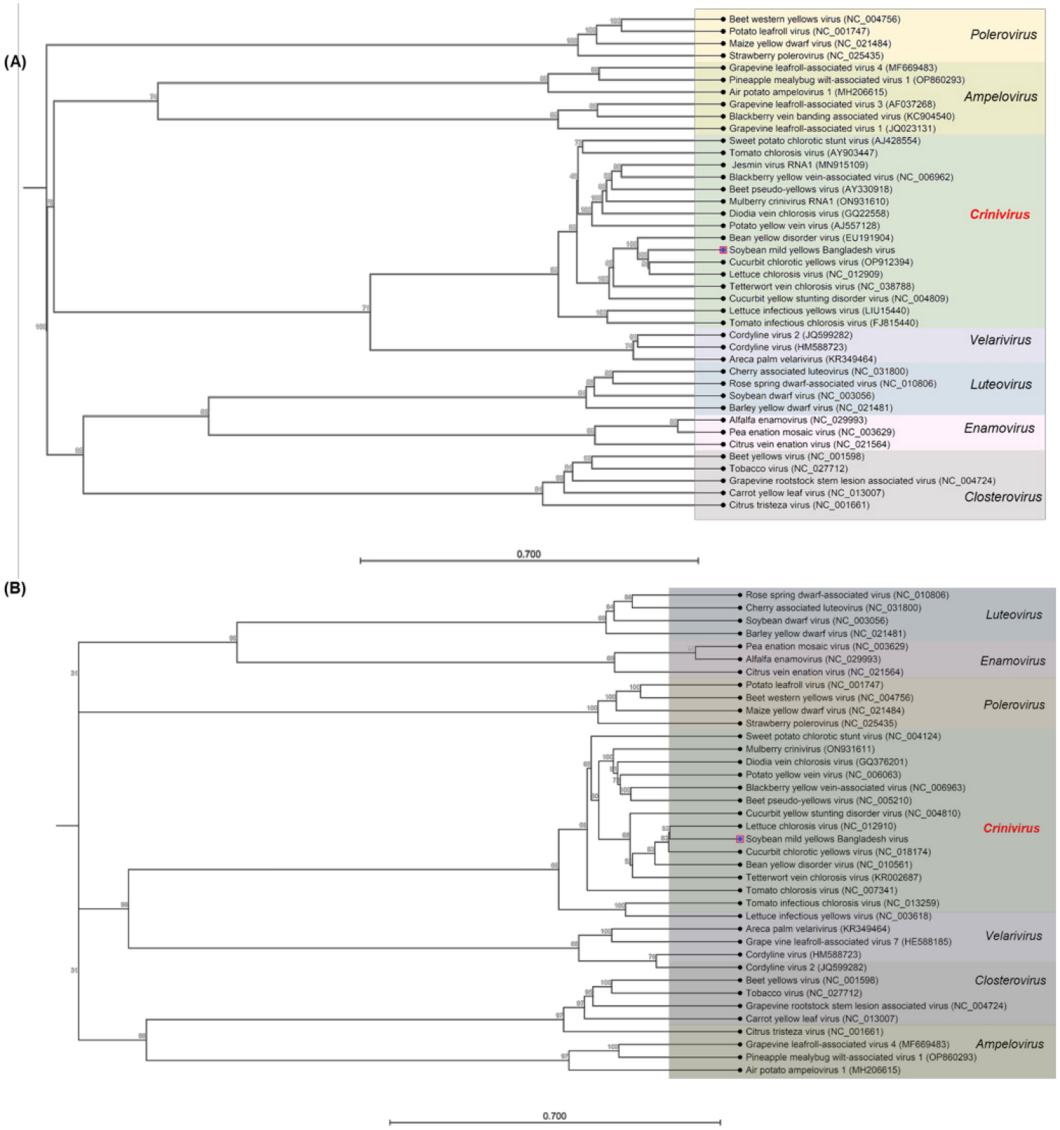
**(A,B)** Phylogenetic trees showing the relationship between the soybean mild yellows Bangladesh virus (SMYBV, RNA1, and RNA2) and other genera were reported in the NCBI GenBank database using the CLC Genomics Workbench. The trees were constructed using the neighbor-joining method and the Jukes–Cantor model with 1,000 bootstraps. The GenBank accession number of each sequence is shown.

Phylogenetic trees were constructed based on the nucleotide sequences to establish relationships between the criniviruses (Figures 3A,C) and other genera (Figures 4A,B). A pairwise nucleotide identity color distance matrix for novel crinivirus (RNA 1 and RNA2) was calculated using SDT v1.2 (Supplementary Figure S4). The amino acid sequences of ORF1a, ORF1a/b, HSP70h, CP, and CPm of SMYBV were analyzed to confirm their relationships with other criniviruses (Supplementary Figure S5). In this study, SMYBV (RNA1 and RNA2) clustered consistently with other viruses of the genus *Crinivirus* and differed from other genera (Figures 4A,B). Phylogenetic analysis confirmed that LCV and CCYV were the closest relatives of SMYBV. Pairwise comparison indicated that 64.92% (OP912394) of RNA1 was associated with CCYV, whereas RNA2 was associated with LCV at 63.24% (NC_012910) (Figures 3B,D). In addition, pairwise comparisons of the amino acid sequences of SMYBV’s highly conserved ORF1a, ORF1a/b containing RdRp, HSP70h, CP, and CPm with those of other criniviruses indicated that SMYBV was most closely related to PloACV, LCV, and CCYV, which shared the highest amino acid identities of 67.63, 71.12, 87.79, 75.29, and 64.93%, respectively (Supplementary Figure S6).

**Figure 5:**
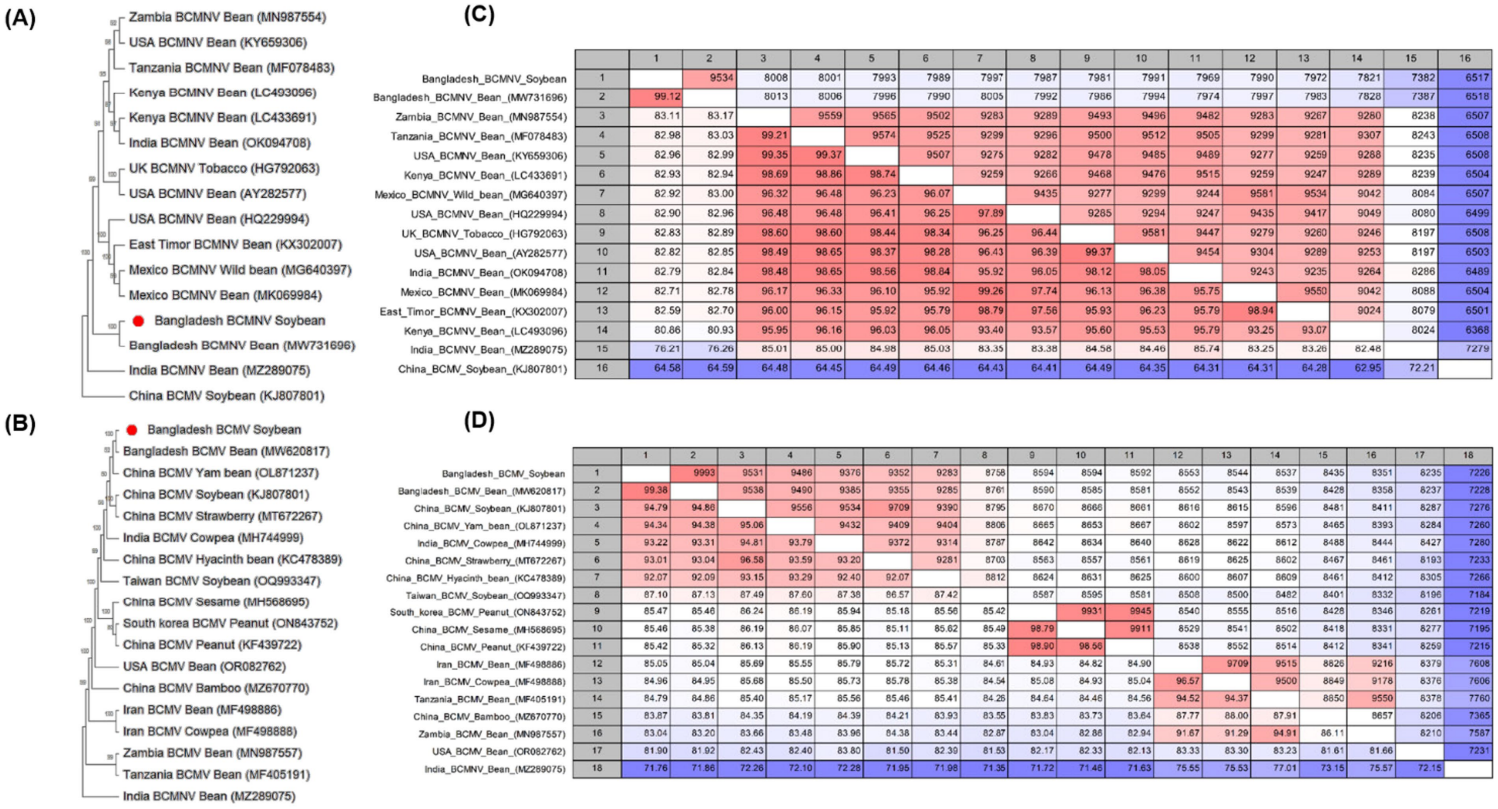
**(A,B)** Phylogenetic trees showing the relationship between *Potyvirus phaseoli* (bean common mosaic necrosis virus, BCMNV) and *P. phaseovulgaris* (bean common mosaic virus, BCMV). The trees were constructed using the maximum likelihood method and the Jukes–Cantor model with 1,000 bootstrap replicates. Evolutionary analyses were conducted using Mega 11. GenBank accession numbers for each sequence are shown. **(C,D)** Pairwise comparisons of BCMNV and BCMV sequences were reported in the NCBI GenBank database using the CLC Genomics Workbench. In pairwise comparisons among the sequences, the upper level showed identity, whereas the lower level showed percentage identity.

Phylogenetic analyses and pairwise comparisons were performed on the nucleotide sequences of MYMIV to confirm their relationship with other MYMIV isolates from the databases of different countries. DNA-A and DNA-B of the MYMIV segments were mostly clustered with NCBI accession numbers OK431079 and MF683073, respectively (Supplementary Figures S8A,B). Furthermore, a pairwise comparison of two sequences of DNA-A revealed that one sequence at 99.60% and the other at 99.20% (OK431079) were associated with MYMIV (India), whereas one sequence of DNA-B was associated with it at 98.57% and another sequence was at 73.17% (MF683073) (Supplementary Figures S8D,E). The sequences of BCMV and BCMNV were used to construct a phylogenetic tree (Figures 5A,B). The analysis revealed that the BCMNV sequence (9,619 nt) showed close similarity to the Bangladesh isolate (MW731696). Comparative analysis indicated a high degree of identity, with 99.12 and 99.35% similarities at the nucleotide and amino acid levels, respectively (Figure 5C). This suggests low sequence variability within BCMNV in Bangladesh. Similarly, the nucleotide sequence (10,055 nt) of BCMV exhibited a 99.38% similarity to Bangladesh isolates (MW620817), with a corresponding 99.35% similarity at the amino acid level (Figure 5D).

### 3.4 Co-infections of novel crinivirus, potyvirus, and begomovirus

RT-PCR and PCR analyses were carried out to assess the presence and coexistence of the novel crinivirus (SMYBV), potyviruses (BCMNV and BCMV), and begomovirus (MYMIV) in soybean plants collected from Gazipur, Bangladesh. Among the collected samples, 19 exhibited clear mosaic and mottling symptoms, 7 displayed mild yellow, and 17 were asymptomatic. Notably, two samples demonstrated co-infection with both SMYBV and MYMIV, while eight samples had single infections of MYMIV, and three samples showed single infections of BCMNV. In the collected samples, a single infection with SMYBV was observed in one asymptomatic plant (Supplementary Table S1). Interestingly, there were no instances of single infections of BCMV noted in this study. In addition, coinfections involving the novel crinivirus, BCMV, and BCMNV were absent. However, MYMIV, BCMV, and BCMNV coinfections were identified in two samples, while MYMIV and BCMNV coinfections were found in two samples. In addition, BCMV and BCMNV coinfections were observed in four samples (Supplementary Table S1). The assessment of virus incidence in the collected samples from the soybean field revealed that the incidence of the novel crinivirus was 6.50% in 2022 and decreased to 3.22% in 2023 (Supplementary Figure S9). The highest incidence of MYMIV was observed in 2023, followed by 2022, in the collected samples. Notably, in 2023, BCMNV had the highest percentage incidence, accounting for 35.48%, among the four viruses assessed.

### 3.5 Seed transmission and sap inoculation test

A total of 36 soybean seedlings were tested for the presence of SMYBV and MYMIV. In this study, no crinivirus or begomovirus infections were observed in the seedlings. Seed transmission tests were conducted for SMYBV and MYMIV, but neither of these viruses was transmitted through seeds. Soybean and tobacco seedlings were mechanically inoculated with sap from symptomatic leaves infected with SMYBV (a novel crinivirus). RT-PCR was performed on the leaves of all the inoculated seedlings using specific primers (Crinivirus_RNA1F/Crinivirus_RNA1R and Crinivirus_RNA2F/Crinivirus_RNA2R) (Table 1). SMYBV was not detected in the inoculated plants in this bioassay (data not shown). In this study, sap inoculation tests for MYMIV, BCMNV, and BCMV were not performed because these viruses are already reported in Bangladesh.

## 4 Discussion

This study aimed to identify viruses associated with virus-like symptoms (Figure 1A; Supplementary Figure S1) observed in soybean plants during 2022–2023. HTS-based sequencing was performed, and subsequent RT-PCR and PCR analyses were conducted on individual samples. Customized bioinformatic workflows were used to identify viruses causing both individual and mixed infections, and alignment-based sequence similarity searches were conducted. The HTS also revealed the presence of bacteria, fungi, arthropods, and oomycetes in soybean samples, using various bioinformatic workflows (Díaz-Cruz et al., 2019). HTS data were used to identify viruses infecting soybeans in Bangladesh and to generate essential molecular information for the development of diagnostic tools for effective virus disease management. In this study, we identified four viruses present in soybean plants in Bangladesh. Using sequencing analysis, we uncovered isolates of viruses from three distinct genera: BCMV and BCMNV within the *Potyvirus genus*, MYMIV in the *Begomovirus* genus, and a novel crinivirus named SMYBV in the *Crinivirus* genus. These findings highlight the diverse array of viruses that infect soybean plants in Bangladesh.

Several RNA viruses have been identified in soybean samples with symptomatic infections (TSV, AMV, TRSV, SbDV, BPMV, SVNV, and ClYVV) in the USA. However, it is important to note that these data should not be construed as indicative of viral prevalence due to the absence of a systematic sampling strategy (Elmore et al., 2022). Interestingly, despite the common perception of SMV being among the most prevalent soybean viruses (Lu et al., 2010), no samples from Bangladesh were found to contain it. Instead, in 2022 and 2023, a novel crinivirus, SMYBV, was discovered in soybean plants in this study.

In 2023, HTS-based transcriptome data revealed a higher virus incidence compared to previous studies due to the enhanced sensitivity and comprehensive detection capabilities of this technology. HTS allows for the unbiased detection of viral RNA, including those present at low levels, which might be missed by traditional methods such as PCR. This approach enables simultaneous detection of multiple viruses, including novel and divergent strains, without prior knowledge of virus sequences (Costa et al., 2022). In our study, HTS facilitated the identification of a novel *Crinivirus* and increased the recorded incidence of BCMNV, BCMV, and MYMIV. Moreover, HTS provides quantitative insights into virus abundance, helping us assess the severity and prevalence of these viruses in soybean crops.

According to the ICTV species demarcation criteria, the amino acid sequences of relevant crinivirus gene products must differ by more than 25%, and nucleotide sequence identity <91% of begomoviruses and potyviruses have complete ORF sequences that are generally <76% identical in nucleotide sequence and < 82% identical in amino acid sequence. These criteria are used to establish distinctions between viral species within the crinivirus, begomovirus, and potyvirus genera (Fuchs et al., 2020; Brown et al., 2015; Fiallo-Olivé et al., 2021; Inoue-Nagata et al., 2022). In this study, SMYBV shared amino acid sequence identity with RdRp (71.12%) of CCYV, HSP70 (87.79%) of LCV, and CP (75.29%) and CPm (64.93%) amino acid sequences with CCYV and PloACV (Table 3). In addition, DNA-A and DNA-B shared 99.60 and 98.57% nucleotide sequence identities with that of MYMIV, respectively (Supplementary Figures S7C,D). Furthermore, BCMV and BCMNV demonstrated a nucleotide sequence identity of over 99% when compared with entries in the NCBI database (Figures 5C,D).

Criniviruses are typically categorized into three groups based on their genomic organization, host range, and transmission characteristics. SMYBV, as a bipartite crinivirus, shares key features with other members of the *Crinivirus* genus, including the presence of RNA1 and RNA2 segments, each encoding multiple open reading frames (ORFs). However, the unique sequence identity of SMYBV, particularly in its HSP70h, CP, and RdRp proteins, places it distinctively within the *Crinivirus* genus, separate from the known groups. Our findings indicate that SMYBV demonstrates significant divergence in nucleotide and amino acid sequences from closely related criniviruses such as CCYV, LCV, and PloACV, suggesting that it may represent a novel species within the genus. This further supports our proposal for classifying SMYBV as a new species within *Crinivirus*. The information we provide may be useful for modifying the current criteria for species demarcation. In addition to amino acid identifiers, host plants of the virus, segments, and transmission vectors should be considered. We have proposed SMYBV as a new species in the *Crinivirus* genus, reflecting its substantial genetic divergence.

SMYBV had bipartite, positive-sense, single-stranded RNA genomes, similar to other criniviruses. The SMYBV genome was sequenced. Three ORFs were identified in RNA1, and seven ORFs were identified in RNA2. The cucurbit chlorotic yellow virus (CCYV) is a bipartite crinivirus that encodes four RNA1 proteins and eight RNA2 proteins (Okuda et al., 2010). Like other members of the crinivirus, the lettuce chlorosis virus has a 17-kb genome encoding four proteins in RNA1 and 10 proteins in RNA2 (Salem et al., 2009). The tomato chlorosis virus (ToCV) genome is 16.8 kb in size and encapsulates long, flexuous virions approximately 800–850 nm in diameter (Liu et al., 2000). RNA1 encodes four ORFs, including proteins involved in viral replication and gene silencing suppression (Wintermantel et al., 2005; Cañizares et al., 2008), and RNA2 encodes nine ORFs that perform a variety of functions, including viral encapsulation, cell-to-cell movement, membrane association, and whitefly transmission (Stewart et al., 2010; Chen et al., 2011).

SMYBV was detected in the leaves of asymptomatic and symptomatic soybean plants, respectively. In the present study, soybean plants were co-infected with SMYBV and MYMIV. The ability of criniviruses studied to date to interact with other viruses in plants and alter their symptoms is one of their most interesting characteristics. According to previous studies, host-specific competition between crinivirus species influences the accumulation of other viruses within the plant and, hence, the severity of symptoms (Karyeija et al., 2000; Susaimuthu et al., 2008; Wintermantel et al., 2008). Mungbean yellow mosaic India virus (MYMIV) was previously reported in Bangladesh. In our study, we found that MYMIV can coexist with the novel soybean mild yellows Bangladesh virus (SMYBV), which may have a more significant impact on soybean production in Bangladesh. Mixed infections of viruses, such as MYMIV and SMYBV, could lead to enhanced symptom severity or altered viral dynamics, which may impact soybean yield and quality in Bangladesh. Host-specific competition between these viruses might influence their accumulation within the plant, exacerbating disease symptoms and potentially increasing the challenge for effective management.

There are concerns about the potential infection of pulse crops in Bangladesh by various viral diseases, such as MYMIV, MYMV, LCV, BYMV, AMV, and PLRV. Different plant viruses in pulse crops have been provisionally identified based on symptoms and serological tests (Akhter et al., 2019). Before our discovery, there was no documented evidence of BCMV and BCMNV viruses infecting soybean crops in Bangladesh. However, our recent findings reveal that soybeans in Bangladesh have indeed been infected with both potyviruses (BCMV and BCMNV). Previously, BCMV and BCMNV were reported to infect common beans in Bangladesh. HTS has also been utilized to detect viruses in soybeans and other crops in Korea. Eight significant viruses—SMV, SYMMV, SYCMV, PeMoV, BCMV, BCMNV, PSV, and TSWV—were detected in soybeans in Korea using HTS (Jo et al., 2020). HTS analysis has been utilized to examine the viral diversity associated with common bean (*Phaseolus vulgaris* L.) in India. Three viruses were identified: BCMV, BCMNV, and clover yellow vein virus (ClYVV), with BCMV being more widespread. In addition, BCMNV and ClYVV were reported as new records from India (Rashid et al., 2022).

To investigate the mechanical transmission of SMYBV, the sap from infected leaves was mechanically inoculated into *Glycine max* (soybean seedlings). However, SMYBV was not mechanically transmissible, which is consistent with previous studies indicating that criniviruses are phloem-restricted and not readily transmitted through mechanical inoculation (Kiss et al., 2013). Attempts were also made to inoculate *Nicotiana tabacum* (tobacco plants) with SMYBV and to amplify its genome using rolling circle amplification (RCA). Unfortunately, these approaches did not yield successful results. These outcomes highlight the limitations of mechanical inoculation and RCA for this particular virus. While our study relied primarily on HTS-based techniques to identify and characterize SMYBV, future research could benefit from using additional techniques such as serological assays, host range studies, or agroinoculation to further elucidate the transmission dynamics and host specificity of this novel crinivirus.

Vectors play pivotal roles in the transmission and spread of viruses over long distances (Samarskaya et al., 2023). Aphids transmit potyviruses, while begomoviruses are exclusively transmitted by *Bemisia tabaci*. Crinivirus transmission is species-specific and carried out exclusively by whiteflies, including those belonging to the *Trialeurodes* and *Bemisia* genera. Future research endeavors should focus on assessing the prevalence of insect vectors and investigating the occurrence of novel and other viruses in soybean crops in Bangladesh.

The detection of the presence of this novel virus in soybean samples could enable us to determine its impact on soybean crops in Bangladesh. As a result, we would be able to gain a better understanding of viral diversity in soybean crops, assisting disease management and crop protection strategies. Implementing measures such as crop rotation, vector control, and phytosanitary practices may prove effective in reducing the detrimental effects of plant viruses on soybean yields, alongside the development of resistant soybean varieties. Future research should focus on investigating different types of mixed infections in plants, their occurrence patterns, and the diseases that have the greatest potential to affect crop production.

## 5 Conclusion

This study presents a comprehensive investigation into viruses associated with virus-like symptoms observed in soybean plants during 2022–2023 in Bangladesh. Based on HTS-based technology and subsequent molecular analyses, four viruses, BCMV, BCMNV, MYMIV, and SMYBV, infecting soybean plants were identified, indicating the diverse array of viruses impacting soybean crops in Bangladesh. Furthermore, our study proposes the classification of SMYBV as a distinct novel species within the genus *Crinivirus*, marking the first detection of SMYBV in soybeans. Thus, it is necessary to conduct a systematic and extensive survey to identify viruses infecting soybeans. This information could facilitate understanding the interactions of SMYBV with other viruses in soybeans and the development of comprehensive disease management strategies to prevent the further spread of these viruses.

## Data Availability

The datasets presented in this study can be found in online repositories. The names of the repository/repositories and accession number(s) can be found in the article/Supplementary material.

## Footnotes

^1^ https://www.ncbi.nlm.nih.gov/orffinder/
