# Supplementary material for: New insights into viral threats in soybean (Glycine max) crops from Bangladesh, including a novel crinivirus
Source: Front Microbiol. 2025 Feb 18;16:1523767. doi: 10.3389/fmicb.2025.1523767 (PMC11876400; doi:10.3389/fmicb.2025.1523767)
Supplement: Supplementary file 1 [file Data_Sheet_1.zip › Khatun et al_Supplementary figures_20240401/Khatun et al_Supplementary data.pdf]

Supplementary Data

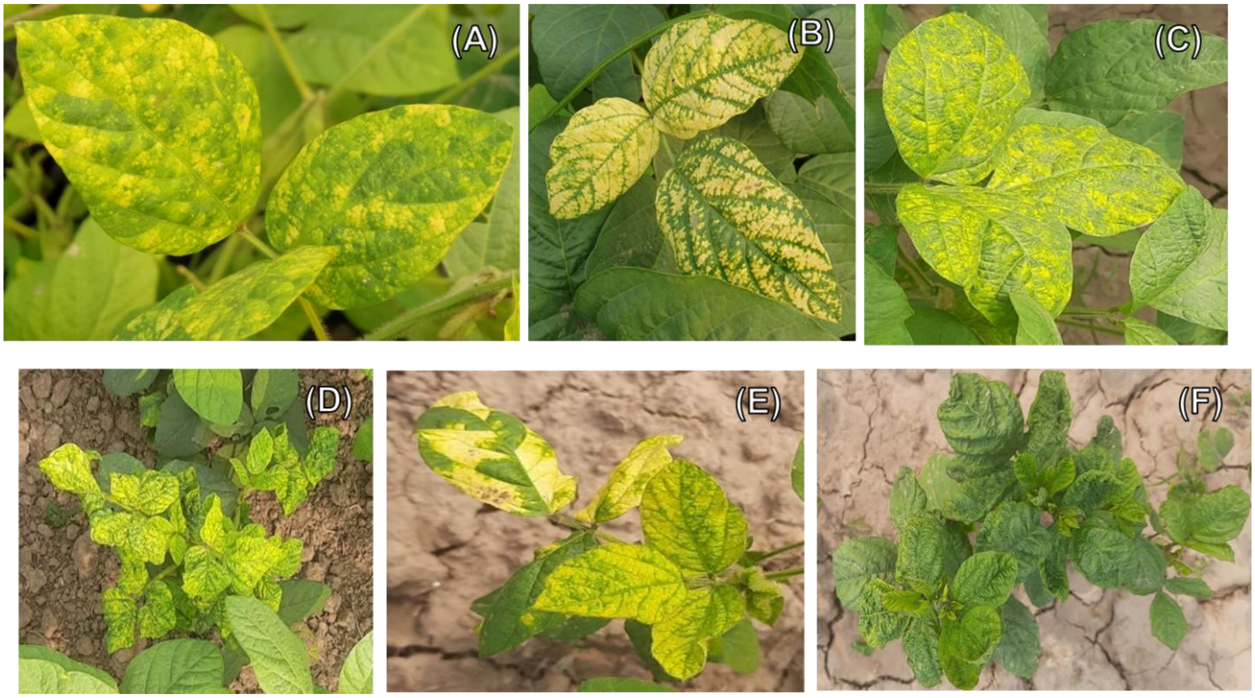

Supplementary Figure S1. Soybean leaf samples showing viral disease symptoms such green or yellow mosaic, leaf wrinkling and mild yellowing leaves were collected from Bangladesh for soybean virome study.

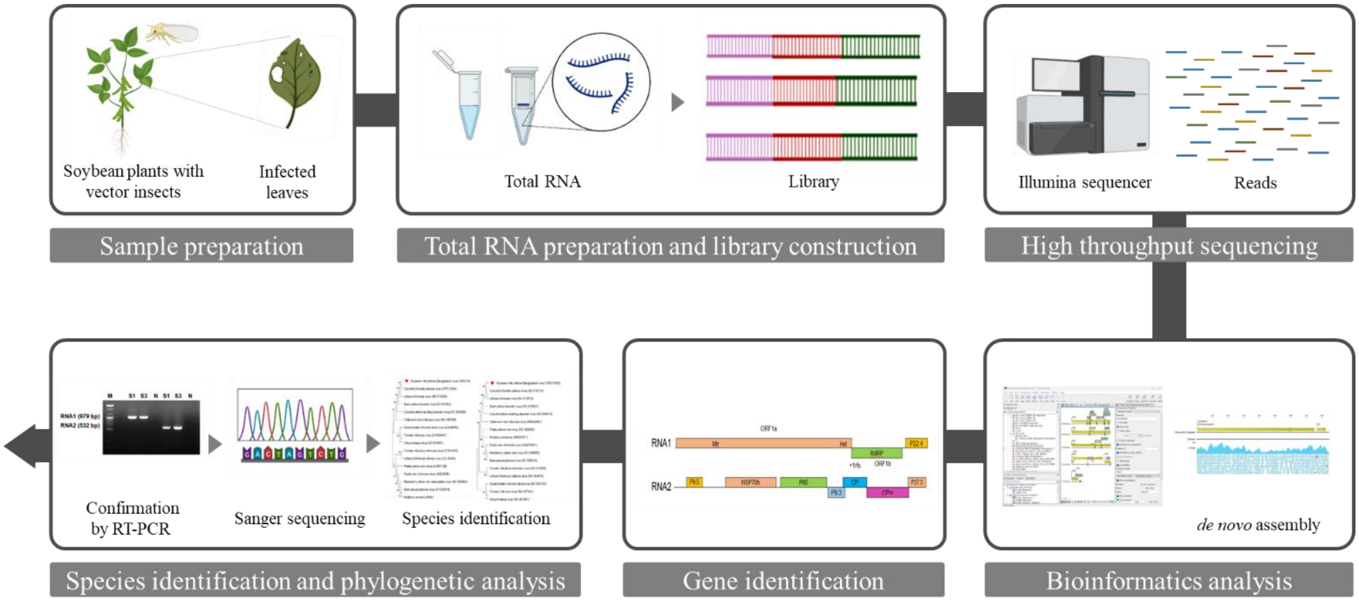

Supplementary Figure S2. Workflow for virus detection and discovery in soybeans using HTS.

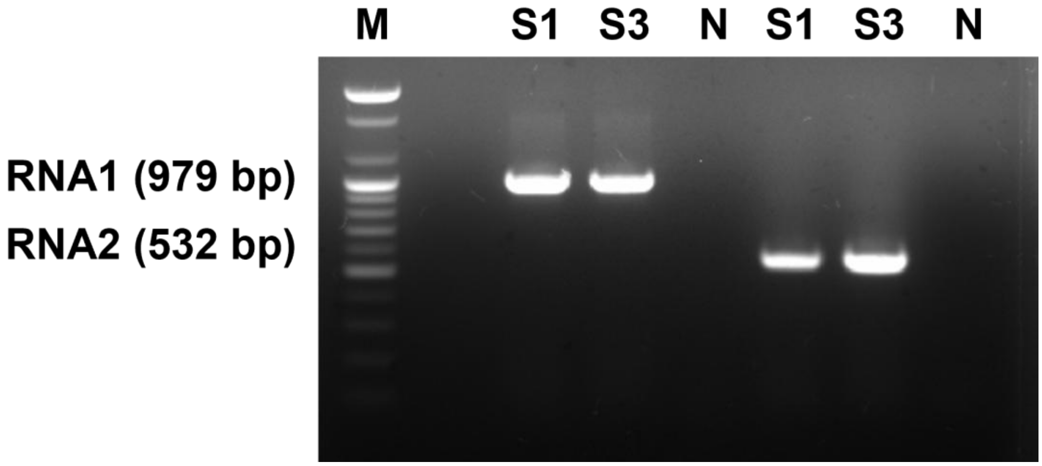

Supplementary Figure S3. Detection of soybean mild yellows Bangladesh virus (SMYBV) infected soybean plants by RT-PCR in 2022-2023. Agarose gel electrophoresis of RT-PCR confirmed the SMYBV presence identified by RNA sequencing. Lane 1: 100 bp DNA ladder (M), Lane 2: negative control (no RNA added), Lane 3-4: Sample 1 and 3 for RNA1 (979 bp), Lane 5: negative control, Lanes 6–7: Sample 1 and 3 for RNA2 (532 bp).

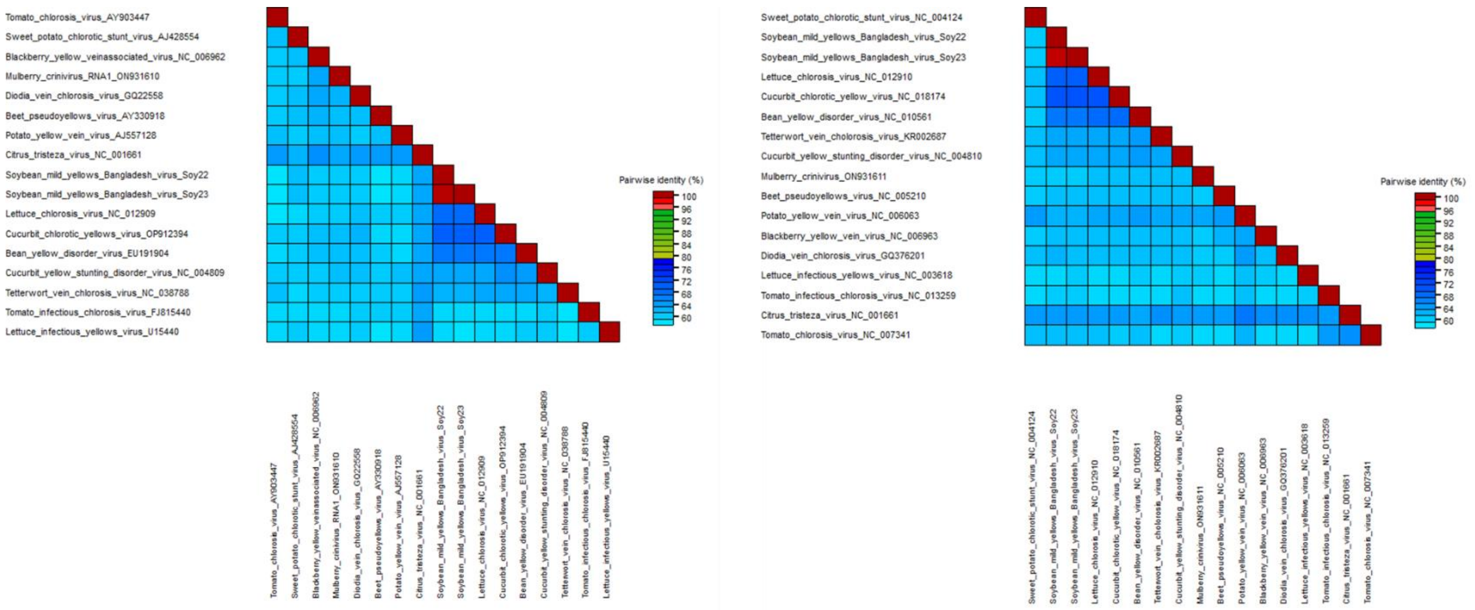

Supplementary Figure S4. Pairwise nucleotide identity color distance matrix for novel crinivirus (RNA 1 and RNA2) calculated by SDT v1.2.

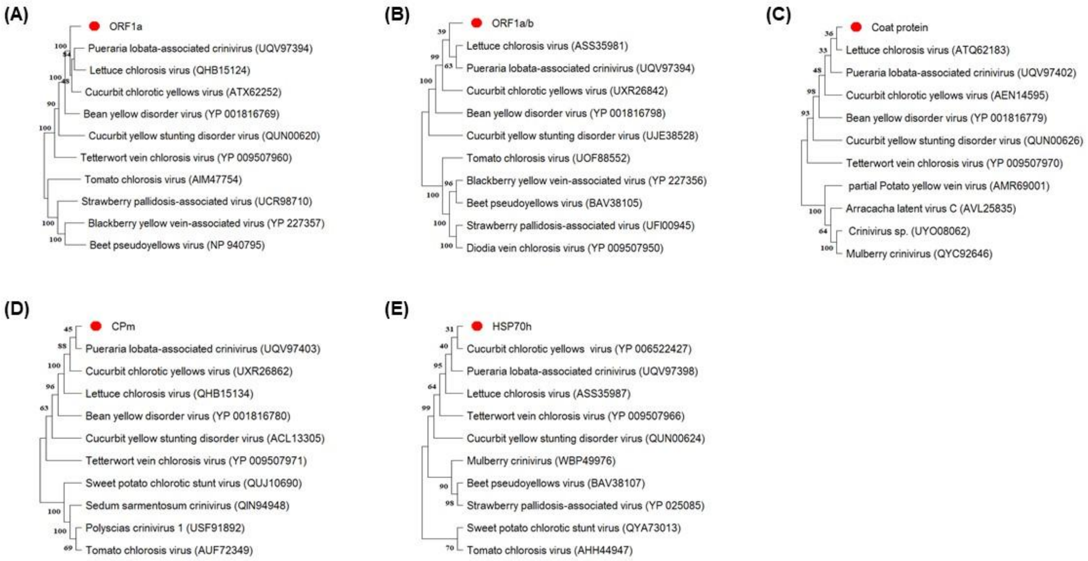

Supplementary Figure S5. Phylogenetic trees were inferred using amino acid sequences of the putative ORF1a, ORF1a/b, coat protein (CP), CPm, and HSP70h (A-E). Phylogenetic trees were constructed using the maximum likelihood method and Jukes-Cantor model with 1000 bootstraps. Evolutionary analyses were conducted in Mega 11. The GenBank accession number of each sequence is shown.

(A)

		1	2	3	4	5	6	7	8	9	10	11
ORF1a	1		1352	1301	1305	1176	986	970	714	685	705	708
CCYV (ATX62252)	2	67.63		1326	1340	1210	986	964	698	685	688	709
PloACV (UQV97394)	3	51.94	52.87		1320	1159	976	953	706	708	1000	694
LCV (QHB15124)	4	65.41	66.83	52.65		1171	965	946	710	702	686	680
BYDV (YP_001816769)	5	57.76	59.28	45.52	57.46		961	984	680	671	687	694
TVCV (YP_009507960)	6	48.98	49.15	38.75	47.89	46.86		884	715	698	715	706
CYSDV (QUN00620)	7	48.23	47.94	37.88	46.99	47.98	43.94		675	649	691	682
BYVAV (YP_227357)	8	34.91	34.08	27.62	34.68	32.87	34.91	32.94		972	902	742
BPYV (NP_940795)	9	33.33	33.28	27.61	34.13	32.29	33.98	31.58	49.69		899	708
SPaV (UCR98710)	10	27.61	26.91	39.06	26.84	26.70	27.98	27.01	36.65	36.34		720
TCV (AIM47754)	11	34.89	34.86	27.27	33.48	33.69	34.71	33.45	36.70	35.07	28.51	

(B)

		1	2	3	4	5	6	7	8	9	10	11
ORF1a/b	1		1778	1725	1730	1326	917	1014	986	993	971	994
CCYV (UXR26842)	2	71.12		1791	1771	1335	955	1006	992	968	954	1005
LCV (ASS35981)	3	68.97	71.44		1760	1353	924	1025	1004	972	956	982
PloACV (UQV97394)	4	68.95	70.76	70.06		1331	903	1021	1016	996	962	999
CYSDV (UJE38528)	5	52.45	52.85	53.54	52.69		766	976	970	992	945	988
BYDV (YP_001816798)	6	36.68	38.23	36.93	36.03	30.85		560	558	565	536	557
BYVAV (YP_227356)	7	39.76	39.37	40.16	39.85	38.32	22.71		1327	1238	1224	1053
BPYV (BAV38105)	8	38.70	38.87	39.37	39.70	38.14	22.52	54.01		1231	1204	1024
SPaV (UFI00945)	9	38.79	37.83	37.94	38.83	38.90	23.14	50.14	49.50		1374	1002
DVCV (YP_009507950)	10	37.26	36.54	36.66	36.76	36.33	21.17	48.49	47.36	54.50		993
TCV (UOF88552)	11	39.34	39.87	38.83	39.53	39.50	22.58	41.67	40.63	39.68	38.55	

(C)

		1	2	3	4	5	6	7	8	9	10	11
Coat protein	1		192	187	186	177	147	148	99	98	99	90
CCYV (AEN14595)	2	75.29		194	190	188	157	155	102	102	104	93
LCV (ATQ62183)	3	73.33	76.68		192	182	152	157	97	98	99	91
PloACV (UQV97402)	4	72.94	76.00	75.89		172	152	149	99	98	100	93
BYDV (YP_001816779)	5	69.41	74.31	72.51	67.98		153	156	99	102	99	89
CYSDV (QUN00626)	6	57.42	61.57	59.61	59.61	60.00		148	94	97	105	98
TVCV (YP_009507970)	7	58.04	61.26	62.55	58.89	62.40	58.27		97	102	100	102
Crinivirus sp (UYO08062)	8	36.80	37.64	35.79	36.53	36.53	34.81	36.47		161	123	101
Mulberry crinivirus (QYC92646)	9	35.90	37.50	36.03	36.03	37.50	35.79	38.78	60.75		127	96
ALVC (AVL25835)	10	36.87	38.24	36.40	36.76	36.40	38.75	37.31	46.59	47.57		107
PYVV (AMR69001)	11	35.86	37.20	36.40	37.20	35.60	38.43	42.15	38.70	36.78	40.68	

(D)

		1	2	3	4	5	6	7	8	9	10	11
CPm	1		311	304	291	237	191	184	125	111	107	84
PloACV (UQV97403)	2	64.93		299	270	223	185	186	120	118	105	90
CCYV (UXR26862)	3	63.47	62.55		283	232	197	186	120	115	113	89
LCV (QHB15134)	4	60.75	56.49	58.96		226	190	179	126	106	100	88
BYDV (YP_001816780)	5	49.27	46.46	48.43	46.89		203	189	118	114	108	96
CYSDV (ACL13305)	6	39.54	38.38	41.04	39.42	42.12		182	117	105	95	84
TVCV (YP_009507971)	7	37.78	38.27	38.35	36.68	39.13	37.30		109	114	98	94
SPCSV (QUJ10690)	8	16.43	15.79	15.77	16.58	15.53	15.37	14.32		167	154	91
Polyscias crinivirus 1 (USF91892)	9	14.66	15.63	15.25	14.02	15.14	13.98	15.08	23.39		305	121
TCV (AUF72349)	10	14.23	14.00	15.09	13.32	14.42	12.70	13.07	21.69	44.99		126
SSCV (QIN94948)	11	17.48	18.79	18.62	18.33	20.08	17.61	19.62	13.15	17.85	18.81	

(E)

		1	2	3	4	5	6	7	8	9	10	11
HSP70h	1		489	488	480	429	406	364	367	343	350	334
LCV (ASS35987)	2	87.79		479	481	439	408	362	366	340	350	333
CCYV (YP_006522427)	3	87.61	86.15		475	434	408	366	367	341	359	334
PloACV (UQV97398)	4	86.18	86.51	85.43		430	404	354	363	343	355	346
TVCV (YP_009507966)	5	76.88	78.82	77.92	77.20		388	353	361	343	365	333
CYSDV (QUN00624)	6	72.89	73.38	73.38	72.66	69.30		345	343	337	340	324
BPYV (BAV38107)	7	65.35	65.11	65.83	63.67	63.38	62.27		404	361	355	332
SPaV (YP_025085)	8	65.77	65.71	65.89	65.17	64.81	61.69	72.66		353	352	336
Mulberry crinivirus (WBP49976)	9	61.58	61.15	61.33	61.69	61.58	60.94	65.16	63.49		336	307
SPCSV (QYA73013)	10	62.84	62.95	64.57	63.85	65.53	61.37	64.08	63.31	60.65		338
TCV (AHH44947)	11	59.96	59.89	60.07	62.23	59.78	58.48	59.93	60.43	55.42	61.01	

Supplementary Figure S6. Pairwise comparisons between amino acid sequences (ORF1a, ORF1a/b, Coat Protein (CP), CPm, and HSP70h) of soybean mild yellows Bangladesh virus (SMYBV) and other sequences reported to NCBI GenBank database using CLC Genomics Workbench (A-E). In pairwise comparisons among the sequences, the upper level showed identities, while the lower level showed percent identity.

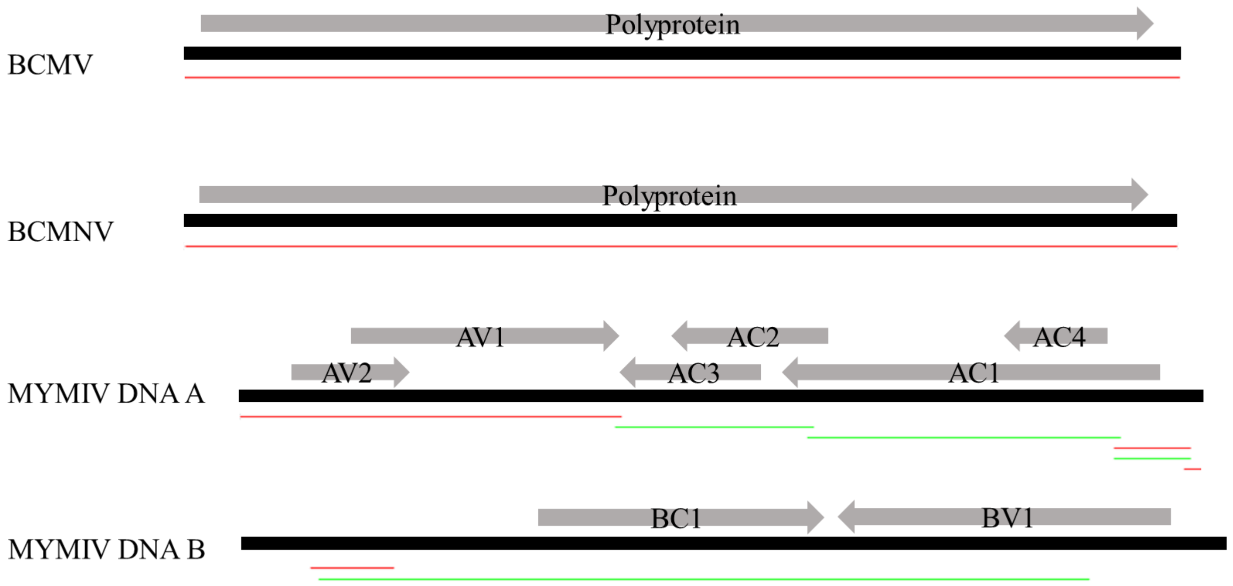

Supplementary Figure S7. Schematic representation of the genome organization of detected viruses bean common mosaic virus (BCMV), bean common mosaic necrosis virus (BCMNV), and mungbean yellow mosaic India virus (MYMIV) from soybeans in Bangladesh. Figures represent ORFs with putative protein products. The red color indicates the assembled contigs of the reverse direction and the green color the forward direction.

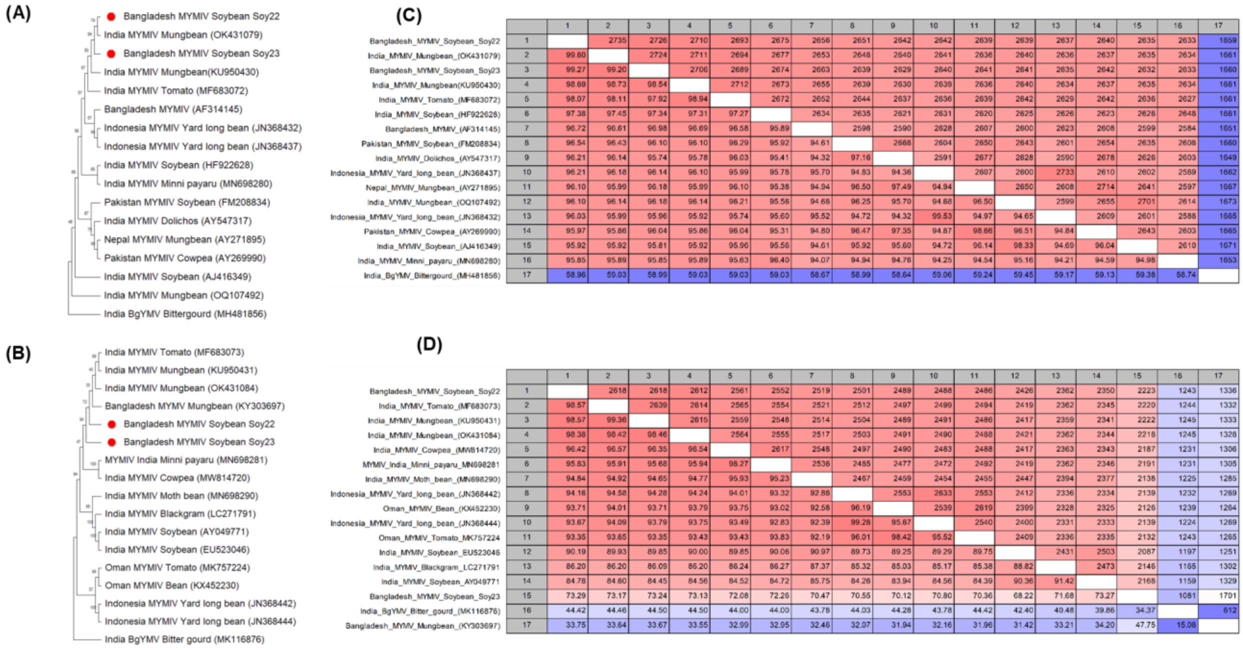

Supplementary Figure S8. (A, B) Phylogenetic trees of mungbean yellow mosaic India virus (MYMIV) were constructed using the maximum likelihood method and the Jukes-Cantor model with 1000 bootstraps. Evolutionary analyses were conducted in Mega 11. The GenBank accession number of each sequence is shown. (C, D) Pairwise comparisons between MYMIV and other complete sequences reported to NCBI GenBank database using CLC Genomics Workbench. In pairwise comparisons among the sequences, the upper level showed identities, while the lower level showed percent identity.

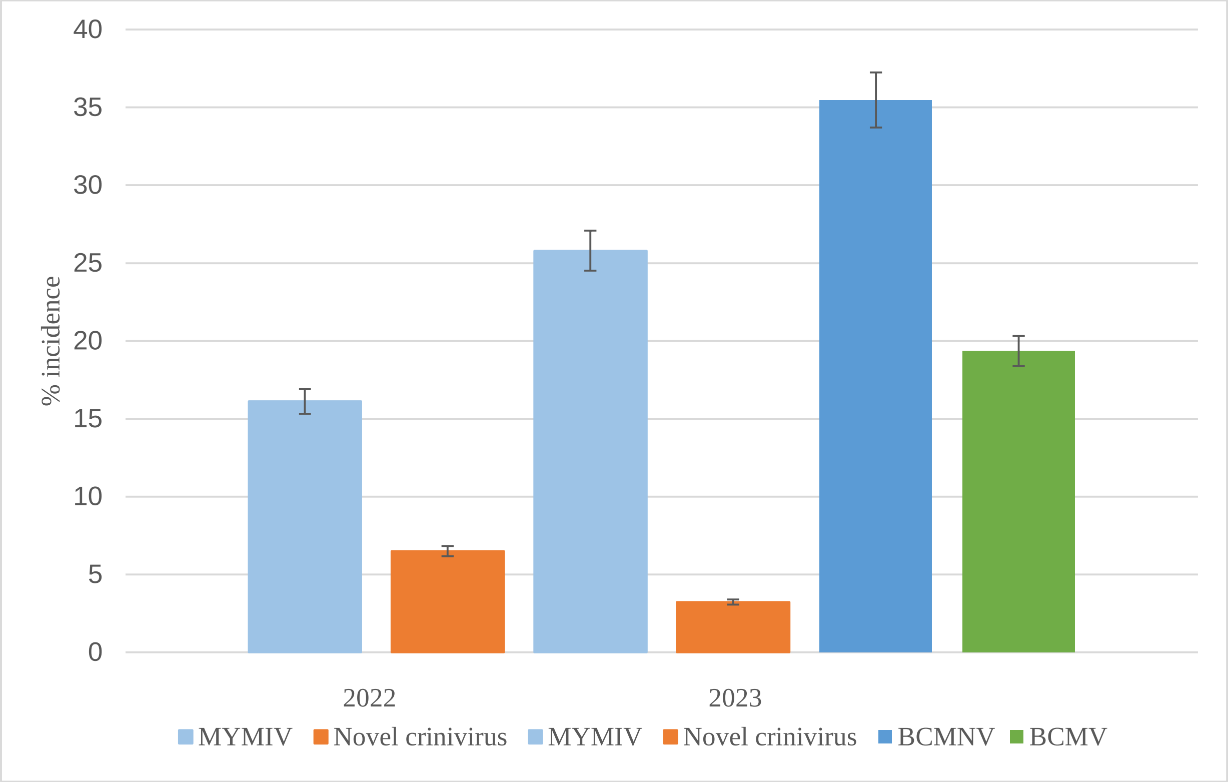

Supplementary Figure S9. Percent incidence of soybean viruses in samples collected from Bangladesh.

Supplementary Table S1

RT-PCR and PCR were used to detect mixed viral infections in soybean leaves from Bangladesh.

Sample no.	Novel crinivirus (Soybean mild yellows Bangladesh virus)		Begomovirus (Mungbean yellow mosaic India virus)		Potyvirus (Bean common mos aic virus)	Potyvirus (Bean common mos aic necrosis virus)	Mixed infection status
	RNA1	RNA2	DNA A	DNA B			
S1	+	+	+	+	-	-	SMYBV+MYMIV
S2	-	-	+	+	-	-	MYMIV
S3	+	+	+	+	-	-	SMYBV+MYMIV
S4	-	-	-	-	-	-	-
S5	-	-	-	-	-	-	-
S6	-	-	+	+	-	-	MYMIV
S7	-	-	+	+	-	-	MYMIV
S8	-	-	-	-	-	-	-
S9	-	-	-	-	-	-	-
S10	-	-	-	-	-	-	-
S11	-	-	-	-	-	-	-
S12	-	-	-	-	-	-	-
S13	-	-	+	+	-	+	MYMIV+BCMNV
S14	-	-	-	-	-	-	-
S15	-	-	-	-	-	-	-
S16	-	-	-	-	-	-	-
S17	-	-	-	-	-	-	-
S18	-	-	+	+	-	-	MYMIV
S19	-	-	-	-	-	-	-
S20	-	-	+	+	-	-	MYMIV
S21	+	+	-	-	-	-	SMYBV
S22	-	-	-	-	-	-	-

S23	-	-	-	-	-	-	-
S24	-	-	-	-	-	-	-
S25	-	-	+	+	-	-	MYMIV
S26	-	-	-	-	-	-	MYMIV
S27	-	-	-	-	-	+	BCMNV
S28	-	-	-	-	-	+	BCMNV
S29	-	-	+	+	+	+	MYMIV+BCMV+BC MNV
S30	-	-	-	-	+	+	BCMV+BCMNV
S31	-	-	+	+	+	+	MYMIV+BCMV+BC MNV
S32	-	-	+	+	-	-	MYMIV
S33	-	-	+	+	-	+	MYMIV+BCMNV
S34	-	-	-	-	+	+	BCMV+BCMNV
S35	-	-	-	-	-	+	BCMNV
S36	-	-	-	-	+	+	BCMV+BCMNV
S37	-	-	-	-	-	-	-
S38	-	-	-	-	+	+	BCMV+BCMNV
S39	-	-	-	-	-	-	-
S40	-	-	-	-	-	-	-
S41	-	-	-	-	-	-	-
S42	-	-	-	-	-	-	-
S43	-	-	-	-	-	-	-
**Total**	**3**	**3**	**13**	**13**	**6**	**11**	**-**

*The meaning of signs: (-) virus not present, (+) virus present; S1-S10 (light orange) samples collected in 2022 and S11-S43 (light blue) collected in 2023.
