# Supplementary figures and images for: New insights into viral threats in soybean (Glycine max) crops from Bangladesh, including a novel crinivirus
Source: Front Microbiol. 2025 Feb 18;16:1523767. doi: 10.3389/fmicb.2025.1523767 (PMC11876400; doi:10.3389/fmicb.2025.1523767)

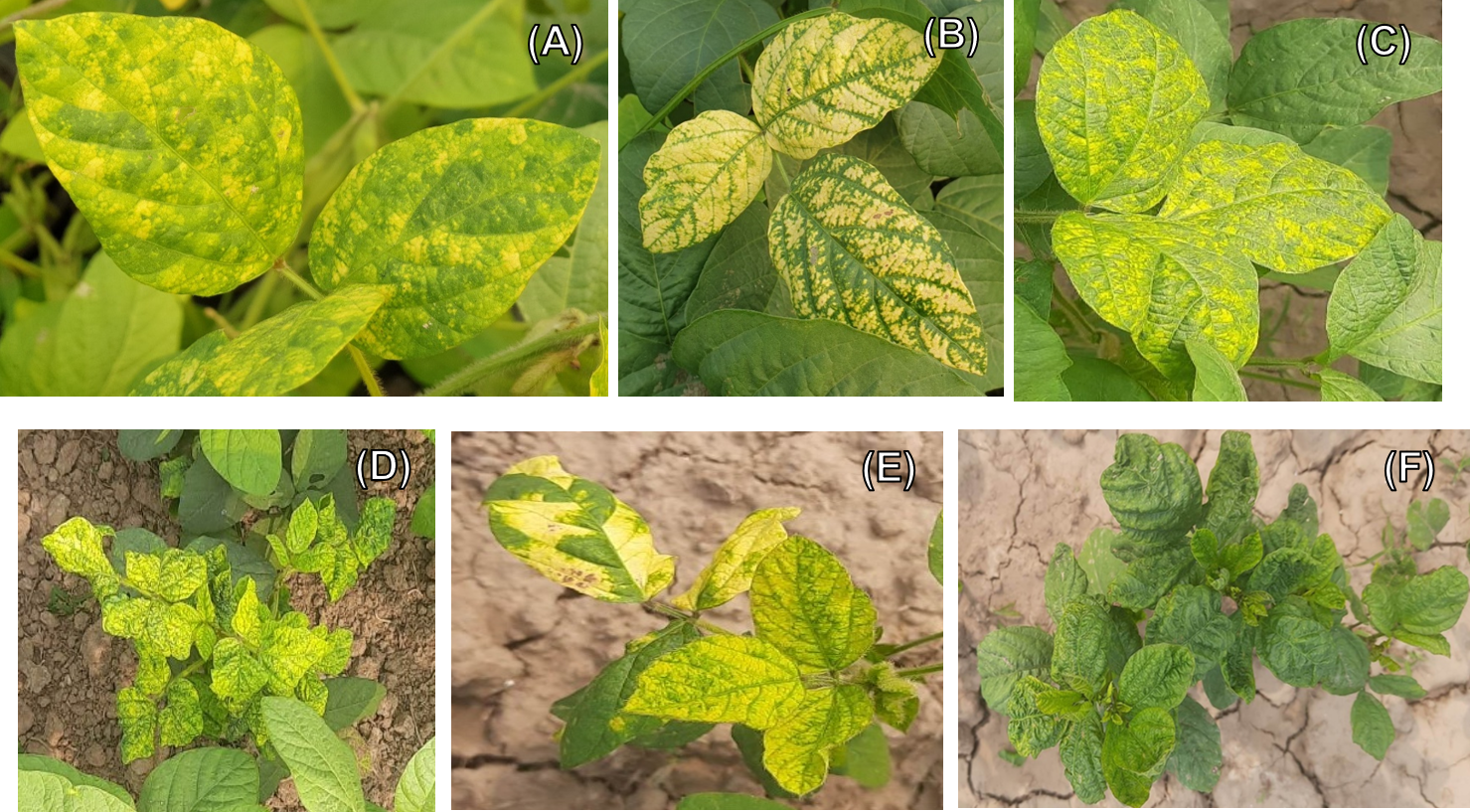

Supplement: Supplementary file 1 [file Data_Sheet_1.zip › Khatun et al_Supplementary figures_20240401/Supplementary Figure 1.tif]

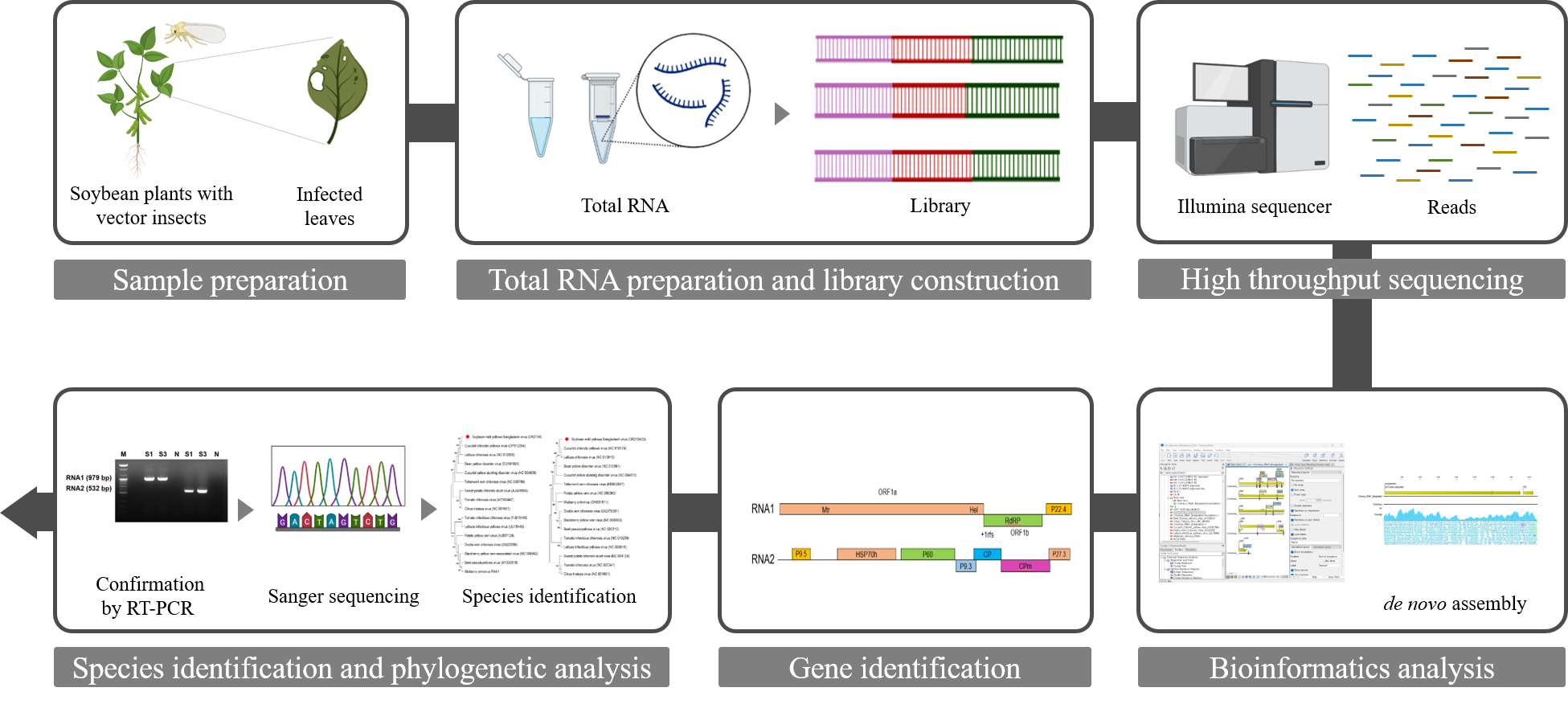

Supplement: Supplementary file 1 [file Data_Sheet_1.zip › Khatun et al_Supplementary figures_20240401/Supplementary Figure 2.tif]

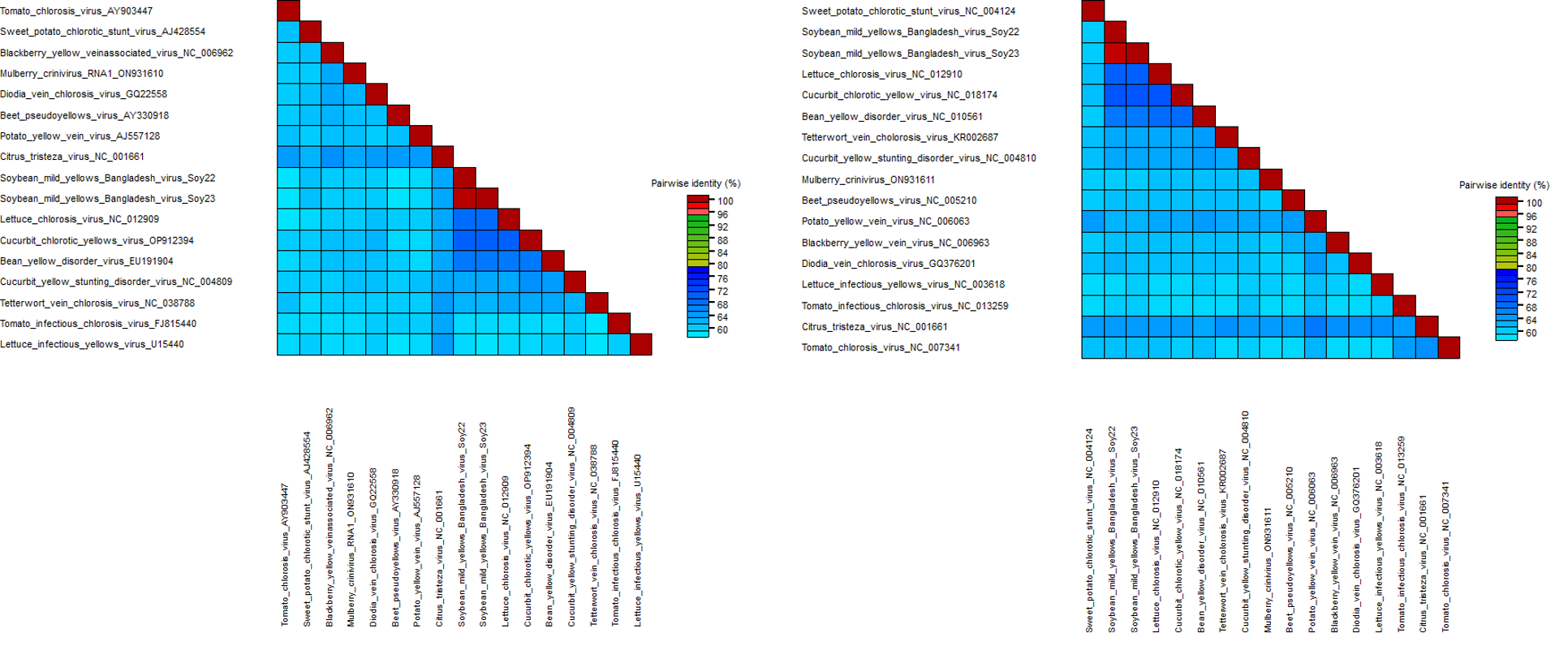

Supplement: Supplementary file 1 [file Data_Sheet_1.zip › Khatun et al_Supplementary figures_20240401/Supplementary figure 4.tif]

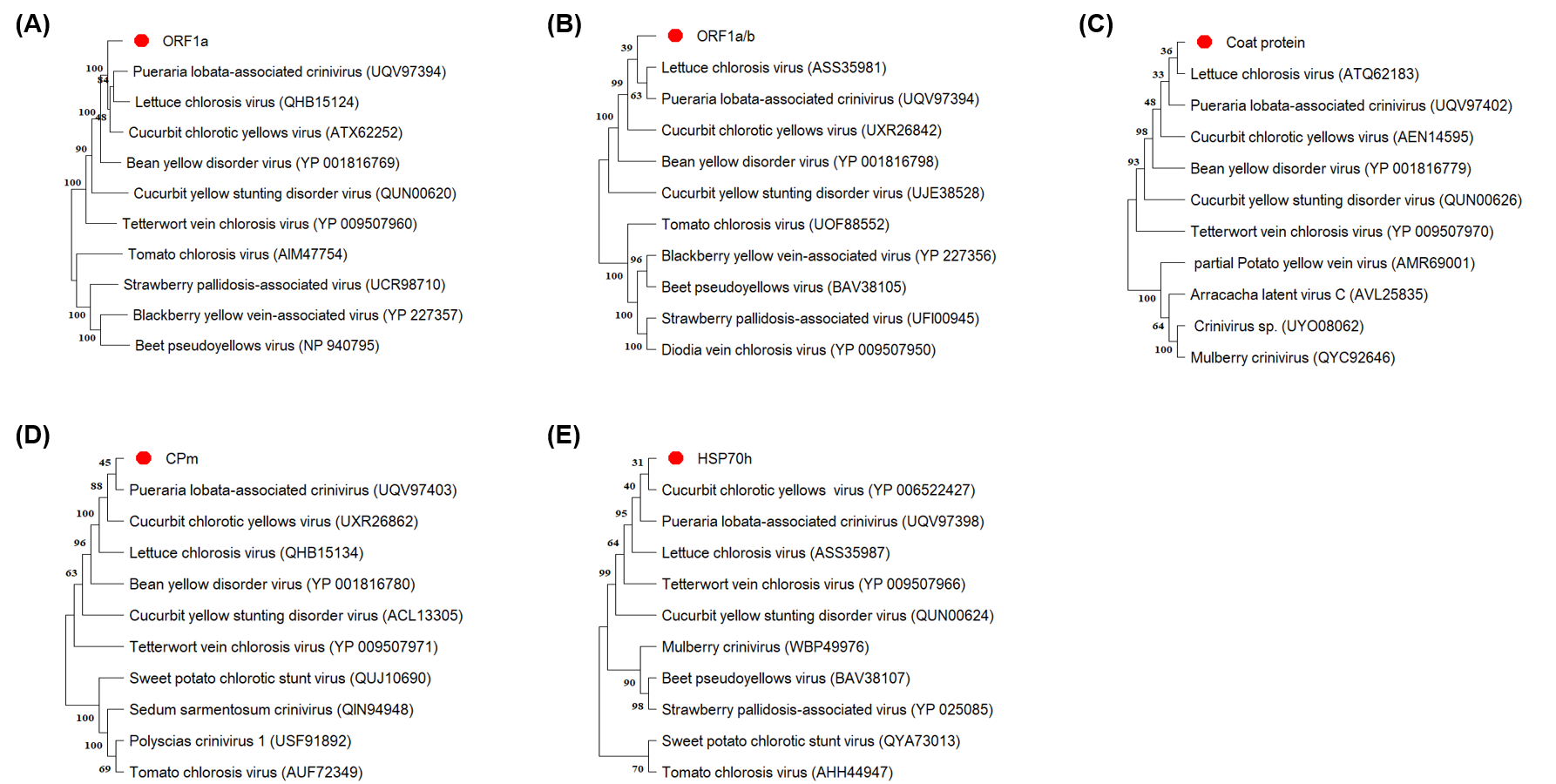

Supplement: Supplementary file 1 [file Data_Sheet_1.zip › Khatun et al_Supplementary figures_20240401/Supplementary Figure 5.tif]

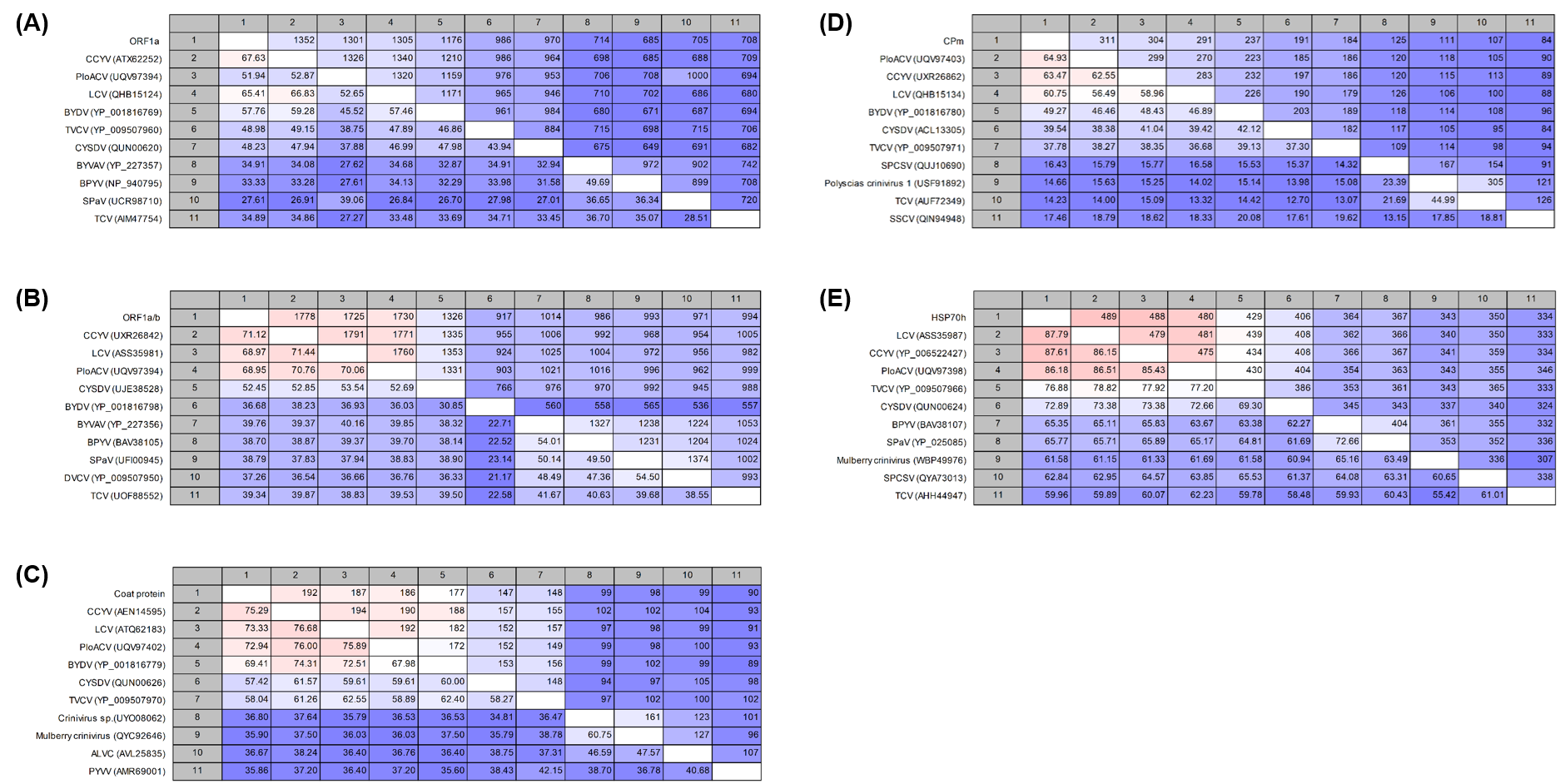

Supplement: Supplementary file 1 [file Data_Sheet_1.zip › Khatun et al_Supplementary figures_20240401/Supplementary Figure 6.tif]

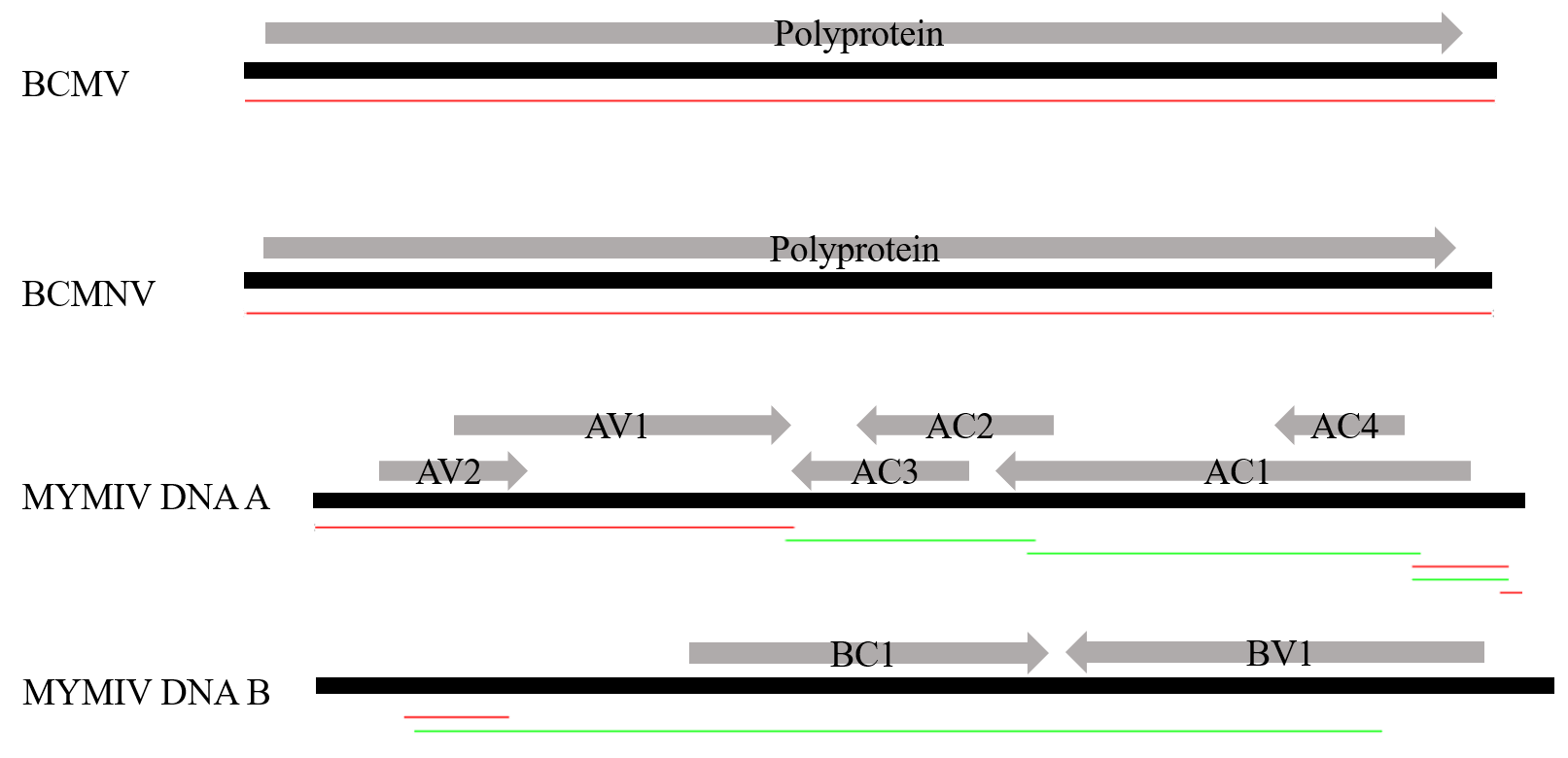

Supplement: Supplementary file 1 [file Data_Sheet_1.zip › Khatun et al_Supplementary figures_20240401/Supplementary Figure 7.tif]

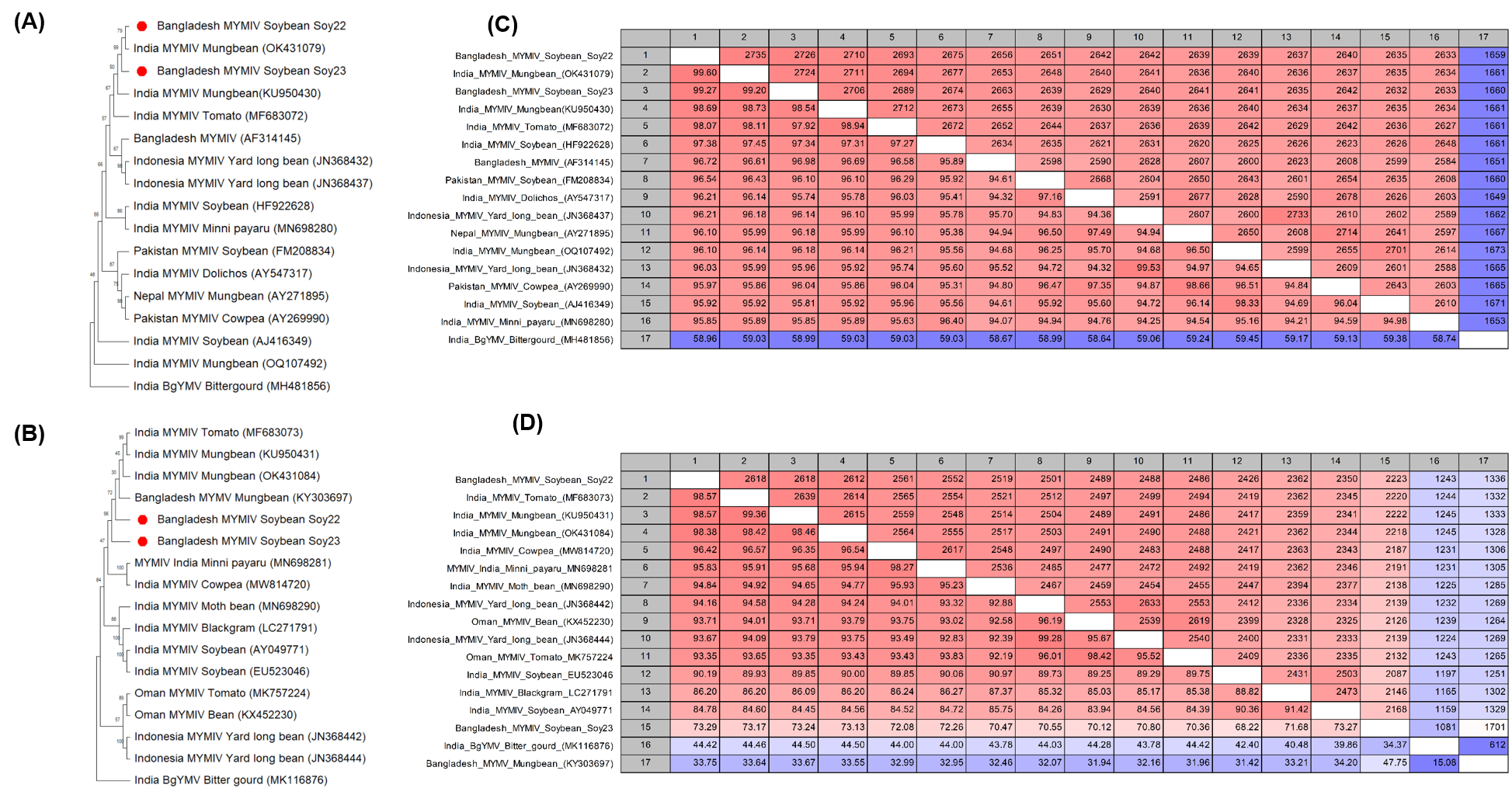

Supplement: Supplementary file 1 [file Data_Sheet_1.zip › Khatun et al_Supplementary figures_20240401/Supplementary Figure 8.tif]

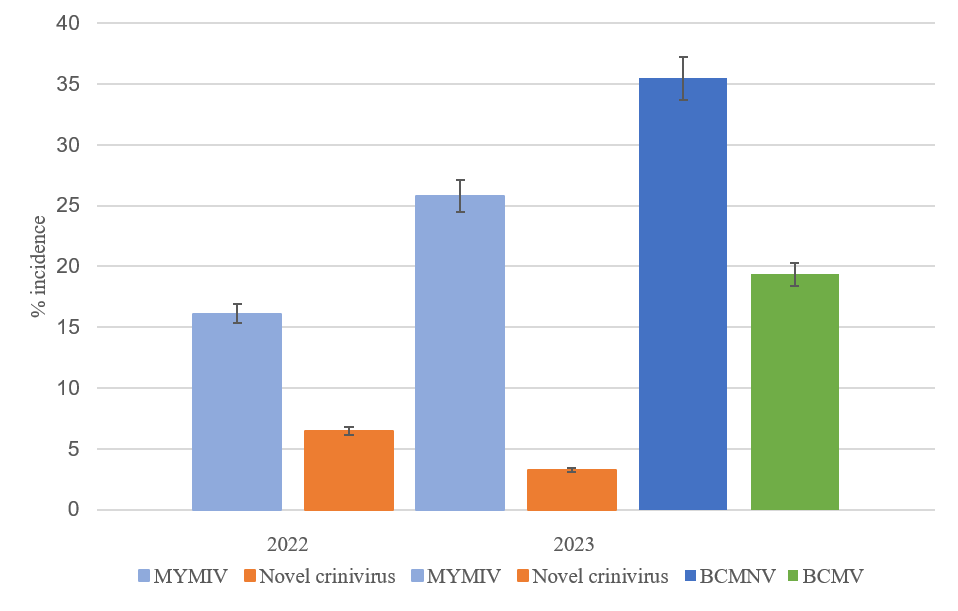

Supplement: Supplementary file 1 [file Data_Sheet_1.zip › Khatun et al_Supplementary figures_20240401/Supplementary Figure 9.tif]

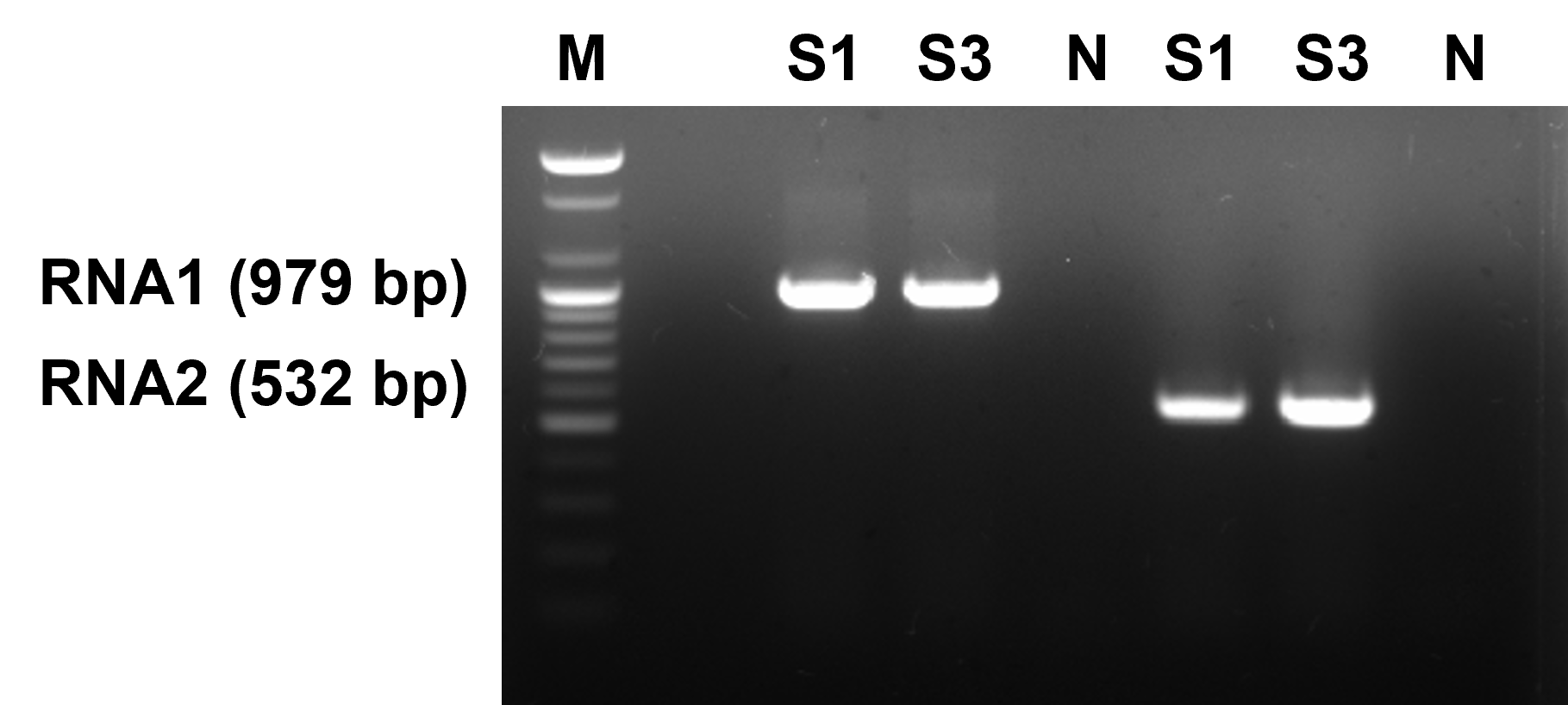

Supplement: Supplementary file 1 [file Data_Sheet_1.zip › Khatun et al_Supplementary figures_20240401/Supplementary Figure 3.tif]
